# FusorSV: an algorithm for optimally combining data from multiple structural variation detection methods

**DOI:** 10.1186/s13059-018-1404-6

**Published:** 2018-03-20

**Authors:** Timothy Becker, Wan-Ping Lee, Joseph Leone, Qihui Zhu, Chengsheng Zhang, Silvia Liu, Jack Sargent, Kritika Shanker, Adam Mil-homens, Eliza Cerveira, Mallory Ryan, Jane Cha, Fabio C. P. Navarro, Timur Galeev, Mark Gerstein, Ryan E. Mills, Dong-Guk Shin, Charles Lee, Ankit Malhotra

**Affiliations:** 1 The Jackson Laboratory for Genomic Medicine, Farmington, CT USA; 2 Department of Computer Science and Engineering, University of Connecticut, Storrs, CT USA; 3 Program in Computational Biology and Bioinformatics, Yale University, New Haven, CT USA; 4 Department of Molecular Biophysics and Biochemistry, Yale University, New Haven, CT USA; 5 Department of Computer Science, Yale University, New Haven, CT USA; 6 Department of Computational Medicine and Bioinformatics, University of Michigan, Ann Arbor, MI USA; 7 Department of Human Genetics, University of Michigan, Ann Arbor, MI USA; 8 The Department of Life Sciences, Ewha Womans University, Seoul, Korea

**Keywords:** Structural variation, Copy number variation, Next generation sequencing, Genome rearrangements

## Abstract

Comprehensive and accurate identification of structural variations (SVs) from next generation sequencing data remains a major challenge. We develop *FusorSV*, which uses a data mining approach to assess performance and merge callsets from an ensemble of SV-calling algorithms. It includes a fusion model built using analysis of 27 deep-coverage human genomes from the 1000 Genomes Project. We identify 843 novel SV calls that were not reported by the 1000 Genomes Project for these 27 samples. Experimental validation of a subset of these calls yields a validation rate of 86.7%. *FusorSV* is available at https://github.com/TheJacksonLaboratory/SVE.

**Electronic supplementary material:**

The online version of this article (10.1186/s13059-018-1404-6) contains supplementary material, which is available to authorized users.

## Background

Structural variations (SVs)—such as deletions, duplications, insertions, inversions, copy number variations, and translocations—are among the most significant determinants of human genetic diversity. Consortium efforts such as the 1000 Genomes Project (1000GP) have recently estimated that a typical genome contains 2100–2500 SVs (> 50 bp), affecting ~ 20 million bp, or ~ 5 times that of SNPs [1]. In contrast to single nucleotide polymorphisms (SNPs), SVs affect large contiguous regions of the genome and they can markedly affect phenotype in many ways, including modification of open reading frames, production of alternatively spliced messenger RNAs, alterations of transcription factor binding sites, structural gains or losses within regulatory regions, and changes in chromatin structure [2]. In addition, it has also been hypothesized that SVs could explain the problem of “missing heritability” from more than a decade of genome-wide association studies (GWAS) into complex human diseases and traits [3, 4]. So far, these studies have relied primarily on commercial SNP genotyping microarrays for identification of causative variants and therefore SVs have been missed from the association tests.

Despite the large scope and potential for critical impact on human biology, SVs remain poorly characterized and understood in human disease primarily due to the lack of comprehensive and robust methods for SV detection. Over the past decade there have been many algorithms developed for detecting SVs using Illumina paired-end short reads (Additional file 1: Figure S1). Careful analyses of these methods have shown the limited overlap amongst the SV calls from these algorithms—primarily because the different algorithms use different strategies (read-depth [RD], paired-end reads [PE], or split reads [SR]) that have different strengths and weaknesses for different types of SVs. To the best of our knowledge, no algorithm comes close to identifying all types of SVs and across all size ranges in human genomes. To overcome this issue, more recently, certain genome consortia studies have combined multiple SV-calling algorithms into a single pipeline to generate a unified SV callset comprising primarily overlapping calls [1, 5]. Methods [6, 7] combining SV calls from multiple algorithms have been previously developed that use either consensus-based or other strategies to obtain a higher quality unified SV callset than what can be obtained from any single algorithm in isolation. However, the optimized way to combine SV calls from multiple algorithms still remains unclear. Simply taking a union of SVs will usually result in a high false-positive rate while taking the overlapping SVs will result in a high false-negative rate. The phenomenon highlights the need to develop an SV detection pipeline which can intelligently combine callsets from multiple algorithms to comprehensively identify SVs in human genomes. Another challenge that currently prevents widespread adoption of the whole genome sequencing (WGS)-based SV detection for disease studies and at the clinic is the lack of robust end-to-end pipelines that will process raw sequencing datasets and comprehensively predict SVs using multiple detection methods. As a solution we present Structural Variation Engine (*SVE*), a computational infrastructure that currently includes an ensemble of eight popular SV calling methods and a novel algorithm—*FusorSV* to merge the SV calls from the ensemble. *FusorSV* employs a data-mining approach to characterize the performance of a group of SV callers against a given truth set. The aim is to select all possible combinations of callers that satisfy a performance threshold.

## Results

### SV detection: *SVE*

**Fig. 1:**
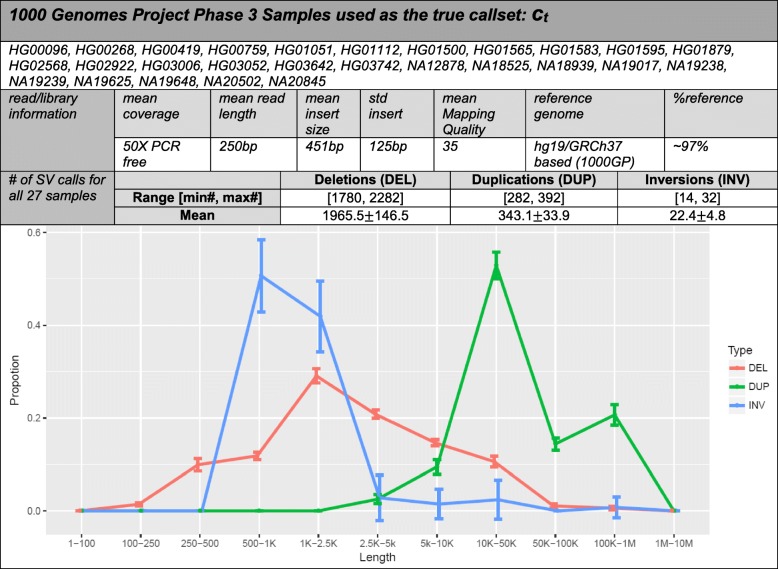
1000GP 27 sample study. 27 samples were selected from the 2504 samples used in the 1000GP due to the availability of high-quality, 50X sequencing coverage comprising polymerase chain reaction-free, 250 bp Illumina PE reads (ftp://ftp.1000genomes.ebi.ac.uk/vol1/ftp/release/20130502/supporting/high_coverage_alignments/20141118_high_coverage.alignment.index). SV types represented in the VCF files were deletions, duplications, and inversions while translocations and other complex SVs were excluded. Mean mapping quality > 30 is considered good. %reference refers to what percentage of the reads mapped to the reference genome

One critical requirement for a successful application of any method at the clinic is robustness and ease of use. To this end, simplified tool installation and proper versioning become minimum requirements for bioinformatics research and need to be addressed in an accelerated and efficient way. To mitigate the problems of tool management, we present *SVE* as a powerful platform that consists of alignment, quality control and the ensemble of eight state-of-the-art SV-calling algorithms (BreakDancer [8], BreakSeq2 [9], cnMOPS [10], CNVnator [11], DELLY [12], GenomeSTRiP [13, 14]^1^, Hydra [15], and LUMPY [7]). *SVE* can be used for any levels of data inputs, such as FASTQs, aligned BAMs, or variant call format (VCFs), and generates a unified VCF as its output (Additional file 1: Figure S2). We then applied *SVE* to the 27 deep-coverage samples of the 1000GP (Fig. 1) [1, 16, 17]. The pipeline starts with BAMs, through eight SV-calling algorithms (including GenomeSTRiP [13, 14] which is not performed via SVE due to the license issues, Additional file 2), and *FusorSV* for a unified VCF for deletions, duplications, and inversions. The running time of each SV-calling algorithm is shown in Additional file 1: Figure S3.

### Ensemble-level SV discovery: *FusorSV*

*FusorSV* provides a unique data-mining method that intelligently takes SV calls from different algorithms and combines them in a manner that minimizes false positives and maximizes discovery. *FusorSV* learns how well different SV-calling algorithms perform compared to a truth set (partitioned by the SV types and sizes which we denote as “discriminating features”) and then applies that information to the decision process. Using per-algorithm performance information and similarity between algorithms, the smallest set of SV callers can be selected using the concept of mutual exclusion, which makes our method both more accurate and comprehensive than other approaches merging SV calls based on consensus or other heuristic. We show that just a consensus from two or more algorithms for a specific SV call does not indicate higher certainty for that event, given that the algorithms used to derive the concordance often share the same underlying assumptions (Additional file 1: Figure S4).

**Fig. 2:**
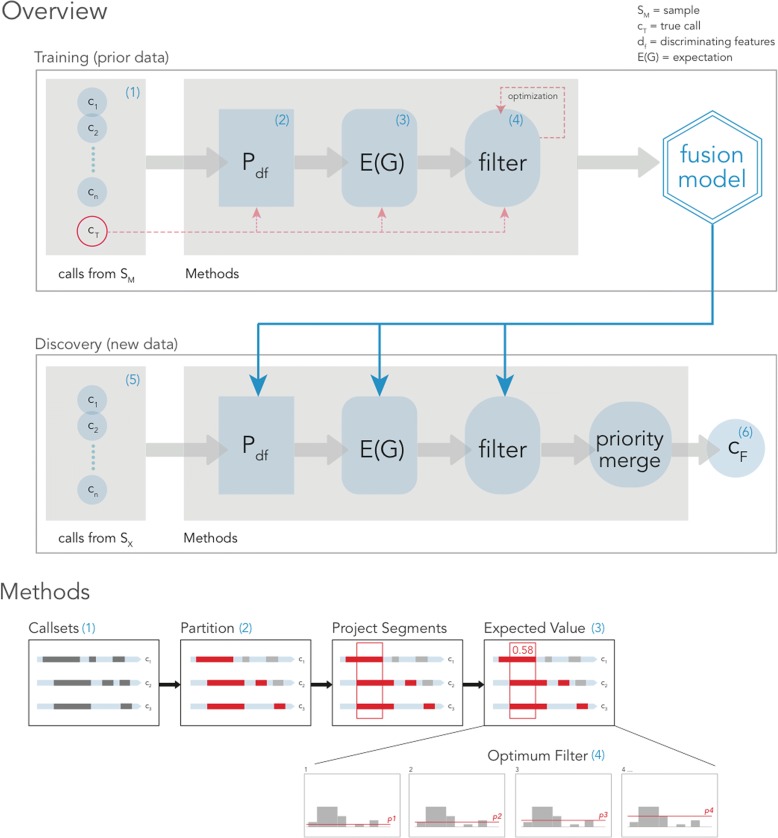
*FusorSV* Framework (see “Methods”). (1) VCF files are first converted to an internal callset representation and then are (2) partitioned using discriminating features. (3) For every partition, a pooled pairwise distance matrix is computed from all observations and then is incorporated into the additive group expectation for every possible combination of callers with Eq. 1 in “Methods.” Partitioned callsets for each sample are projected back into a coordinate single space, where the weight of each disjoint segment is given its previously estimated expectation value by lookup. (4) A partition is fit to the data by returning the value for the proposal expectation cutoff that is the closest to the truth. (5) Given new data during discovery, filtered partitions are merged back together from smallest to largest size, discarding the lesser of overlapping calls by their expectation value and then finally clustered to yield a genotyped VCF output (6)

We use the term “projection” to mark the association of a particular SV call from a particular SV caller to base pairs in the genomic coordinate space (Fig. 2). When calls from two different callers overlap, this information will be marked in the projection in the form of the caller identifier. This creates contiguous segments in coordinate space and it is in these segments that we score the chance of a SV call being correct. These scores can be used effectively to become a filter point for new unseen data. The segments that clear the filter are merged together or discarded if they do not clear the filter. We proceed with two main phases: Training and Discovery (Fig. 2).

#### FusorSV: Training phase (building the model)

*FusorSV* is a method merging callsets from multiple algorithms by a *FusorSV*-trained model. For training a model, *FusorSV* needs a callset from each SV-calling algorithm as well as a truth set of SV calls from one or more sample. Previous studies [7, 18] have shown that SV algorithms can have significant biases in calling specific SV types (i.e. deletions, duplications, and/or inversions) and/or certain SV sizes [7, 18]. Therefore, we use SV type and SV size as the two discriminating features for our training phase. We used a variable number of bins per SV type in the size range of 50 bp–100 Mbp. We then calculated the performance of each of the eight algorithms (including GenomeSTRiP) compared to the truth set, in addition to calculating pairwise performance across all algorithms. We excluded calls < 50 bp or translocations because these SVs were not frequent enough in the truth set to provide enough data to make an informed decision for these cases. SV calls were only considered when the filter value was either unspecified or set to “PASS.” A uniform SV mask mainly comprising regions in the human genome that are known to be difficult to access by current technology and had no intersection with the calls in the truth set was constructed and applied to all algorithms prior to measuring each algorithm’s performance.

For each sample and algorithm, the resulting SV calls were segregated into the bins defined by the conjunction of the two discriminating features (e.g. deletions in the size range 1–5 kb in length would be one bin) (Fig. 2). Once the SV calls from all eight SV-calling algorithms were assigned a bin, we evaluated the performance of all possible combinations of the algorithms by comparing the resulting merged calls in each bin to the truth lying in that same bin. The partitioning scheme is fully discrete implying each call is accounted for in exactly one partition without ties. This step is followed by iterative projection, dynamic filtering, and merging to fit the model which has the effect of generating *FusorSV* output calls that are consistent with the input data and true callset SV features. We used a base-pair Jaccard index [19] (see “Methods”) to evaluate the similarity of a pair of algorithms and the performance of an algorithm against the truth set. When deciding which combination of algorithms is best, two algorithms that are mutually similar are diminished in weight, while more mutually exclusive algorithms are more heavily weighted. This manipulation has the effect of promoting combinations of algorithms that work more comprehensively. This also supports the previous finding that combining two different kinds of evidence, such as a SR and a read pair (RP) increases accuracy [7] and can improve SV detection. The training concludes when the score for every possible combination of algorithms is calculated and the performance value (the similarity between the callset from an algorithm and the truth set) that should be used for decision in each partition is determined. This is what we denote as our *FusorSV* fusion model.

#### FusorSV: Discovery phase (application of the model)

One can use the provided *FusorSV* fusion model built during the training phase for any new sample for a comprehensive SV detection. The first step in the discovery phase involves generating SV calls from the same SV callers used to train the model but on a new sample. SV callsets from the new sample are then treated with the same partitioning scheme and the similarities of pairs of algorithms’ callsets are calculated, however, with no truth set included. As a quality control step, we use a Mantel test [20], to determine whether the input callset is similar to the training model or divergent. Partitioned callsets are projected onto the reference coordinate space forming segments just as in the training phase, while the group expectations are derived from the training model and subsequently filtered using the cutoff value that had maximized performance in training. Segments that are above the cutoff value are joined and the expectation values are averaged proportionally, while segments that are below the cutoff are discarded. When two segments overlap, the segment with the lower expectation is discarded. All remaining segments are sorted by coordinate and the final callset is generated with the expectation value from the model as well as the information of the participating callers.

#### Performance evaluation

To demonstrate the effectiveness of our approach, we performed 1000 rounds of experiments. For each round, 18 samples were randomly chosen from the 27 deep-coverage samples in the 1000GP for the model building. The model was then applied to the remaining nine samples for the performance evaluation. The experiment has been repeated 1000 rounds by different combinations of 18 training and nine testing samples. The truth set used for the training phase was obtained from the 1000GP SV Phase 3 [1, 16] results [21] (see Additional file 3). We also compared our method to MetaSV [6], which is another ensemble method that uses calls from BreakDancer, BreakSeq2, CNVnator, and Pindel [22].

**Fig. 3:**
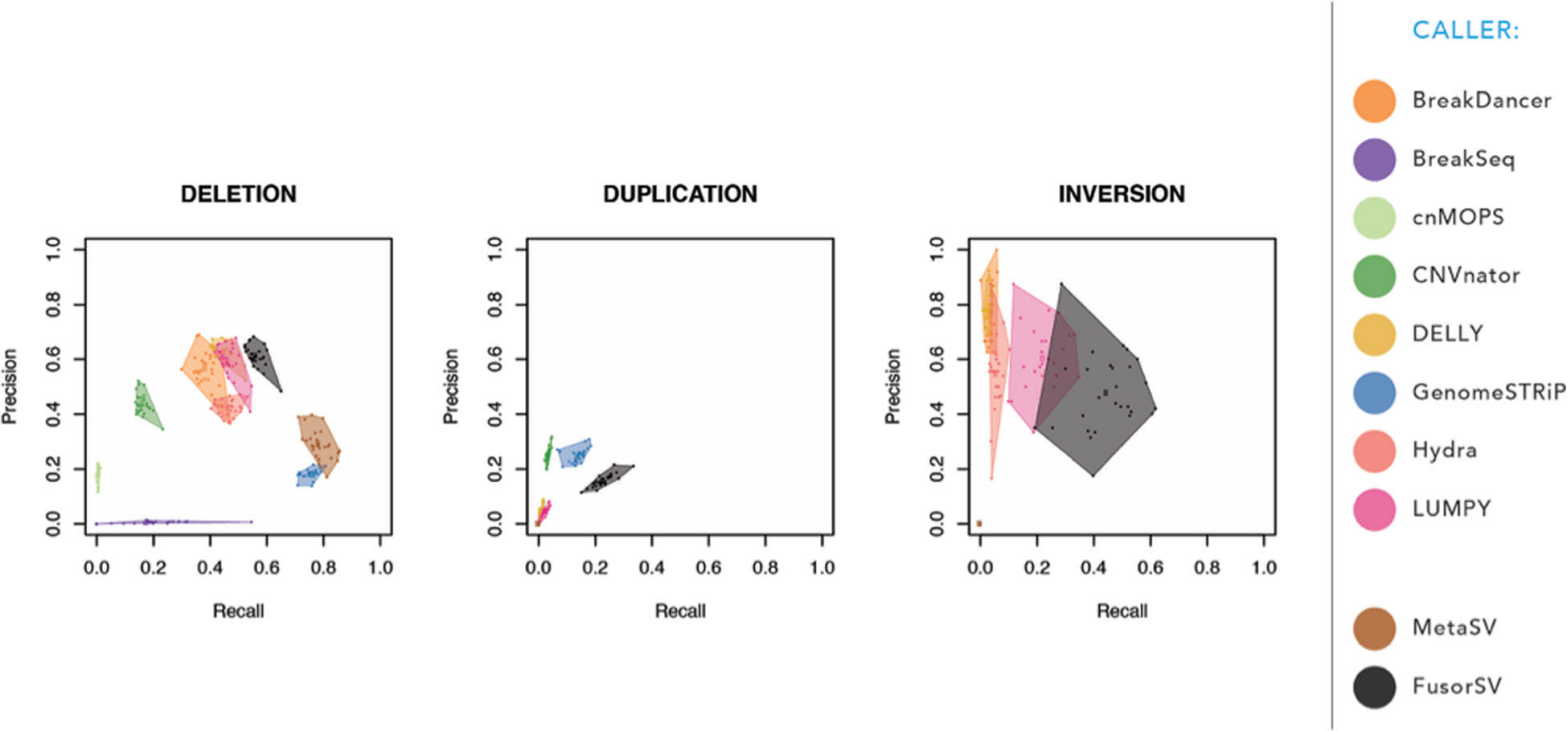
Performance evaluation of *FusorSV*. The results of 1000 rounds of cross-validation where 18 samples were used to train a fusion model and the remaining nine samples were tested. Being closer to the *upper right corner* means better performance, with the *solid dot* depicting the average for all samples. *FusorSV* improves performance by utilizing multiple algorithms while making more total calls than integrative consensus methods like MetaSV

Figure 3 shows the precision (percentage of SVs that overlapped with the truth set over the total number of detected SVs) and recall (percentage of SVs that overlapped with the callset over the total number of SVs in truth set) that are average numbers of 1000 rounds (see “Methods”). We observed that *FusorSV* performed remarkably well by measuring precisions and recalls against the 1000GP Phase 3 SV callset [1, 16]. The complete list of precisions and recalls is provided in Additional file 4: Table S1.

#### Application of 27 deep-coverage samples in the 1000GP

**Fig. 4:**
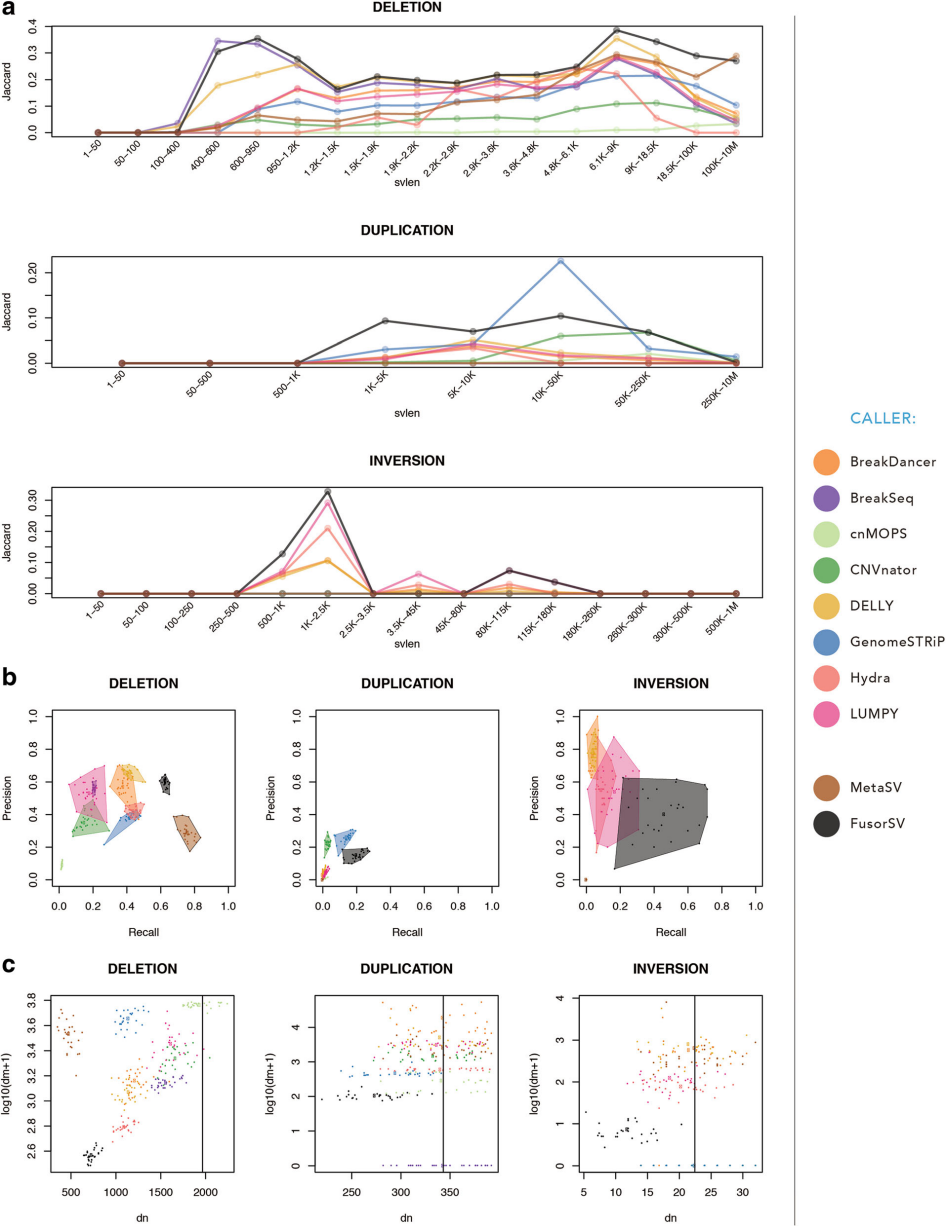
*FusorSV* result of 27 deep-coverage samples. **a** The Jaccard Similarity against the truth set provides the evidence that FusorSV gets more overlaps with the truth set than any single SV-calling algorithm. **b** Precision-recall of all SV-calling algorithms against the truth set. Being closer to the *upper right corner* means better performance, with the *solid dot* depicting the values of a sample. *FusorSV* improves performance by utilizing multiple algorithms while making fewer total calls than integrative consensus methods like MetaSV. **c** Plot depicts number of 1000GP events per sample not called by the specific caller (dm) versus the number of called events not present in the 1000GP (dn). Being closer to the bottom left indicates higher performance. Vertical line denotes average number of calls per sample in 1000GP

We also built a fusion model by applying *FusorSV*/*SVE* to the entire set of 27 samples. The model can be used for any new sequenced sample. For deletion calls, BreakDancer, LUMPY, and DELLY have 57%, 53%, and 66% precision values similar to that for *FusorSV* (60%), but all have lower (38%, 18%, and 42%) recall than *FusorSV* (63%) that negatively impact the F-measure scores (a measure that combines precision and recall, see “Methods”) of BreakDancer, LUMPY, and DELLY. The detail information is shown in Fig. 4 and Additional file 4: Table S2. MetaSV had the highest recall (79%) but was restrictive in calling with precision (29%) and an F-measure (42%). *FusorSV* had high precision and recall, leading to the best F-measure (61%) and a better Jaccard similarity (40%) with the 1000GP Phase 3 SV callset [1, 16, 21]. For Jaccard similarity, *FusorSV* outperformed all individual algorithms (40%) with MetaSV as the next best method (32%), followed by BreakDancer, BreakSeq2, LUMPY, DELLY, and Hydra (all near 20%).

For duplication calls, GenomeSTRiP performed at 26% precision and 14% recall, while CNVnator performed at 22% precision and 3% recall. The MetaSV duplication calls were excluded due to the low quality VCF flag (only single caller support). *FusorSV* had higher recall, F-measure, and Jaccard similarity with the 1000GP Phase 3 SV callset [1, 16, 21], but lower precision (15%) than GenomeSTRiP (26%). This minimal improvement over a single duplication caller supports previous literature with regard to the difficulty of accurate and reliable duplication calling using the Illumina short read sequencing data and a small set of samples [13]. Many duplication calls coming from the PE callers, such as Hydra and LUMPY, seem to have large overlapping transient artifacts, which in general drove down their performance numbers. When repeat regions were excluded, no significant increase in performance was observed.

For inversion calls, DELLY, BreakDancer, Hydra, and LUMPY have high precision values at 79%, 76%, 55%, and 54%, respectively, but they sacrificed recall values (4%, 4%, 9%, and 17%) leading to low F-measure (8%, 8%, 16%, and 25%) and Jaccard similarities (0%, 0%, 1%, and 1%) with the 1000GP Phase 3 SV callset [1, 16]. *FusorSV* (precision = 40%, recall = 58%, F-measure = 44%, and Jaccard similarity = 28%) outperformed the other individual algorithms due to the mutually exclusive information obtained from the combination of DELLY and LUMPY (Additional file 1: Figure S4B). Many more inversion calls were made from algorithms like BreakDancer, compared to the number of inversion calls made in the 1000GP, which guided *FusorSV* to an optimized F-measure and Jaccard similarity. In summary, *FusorSV* outperforms all other individual SV-calling algorithms including the MetaSV ensemble approach across all SV types.

To obtain more data for the training phase of *FusorSV*, we generated 30 simulated human genomes at 50X coverage with simple and disjoint homozygous SVs using Varsim [23]; however, when we compared the simulation data to the real human data, we observed huge disparity in similarity values (Additional file 1: Figure S4A and B), suggesting that the current Varsim simulation framework does not generate realistic human SVs patterns. In contrast, when we looked at other human samples (low coverage 1000GP data), the similarity values were consistent with our 27-sample study (Additional file 1: Figure S4C).

#### In vitro validation results

**Fig. 5:**
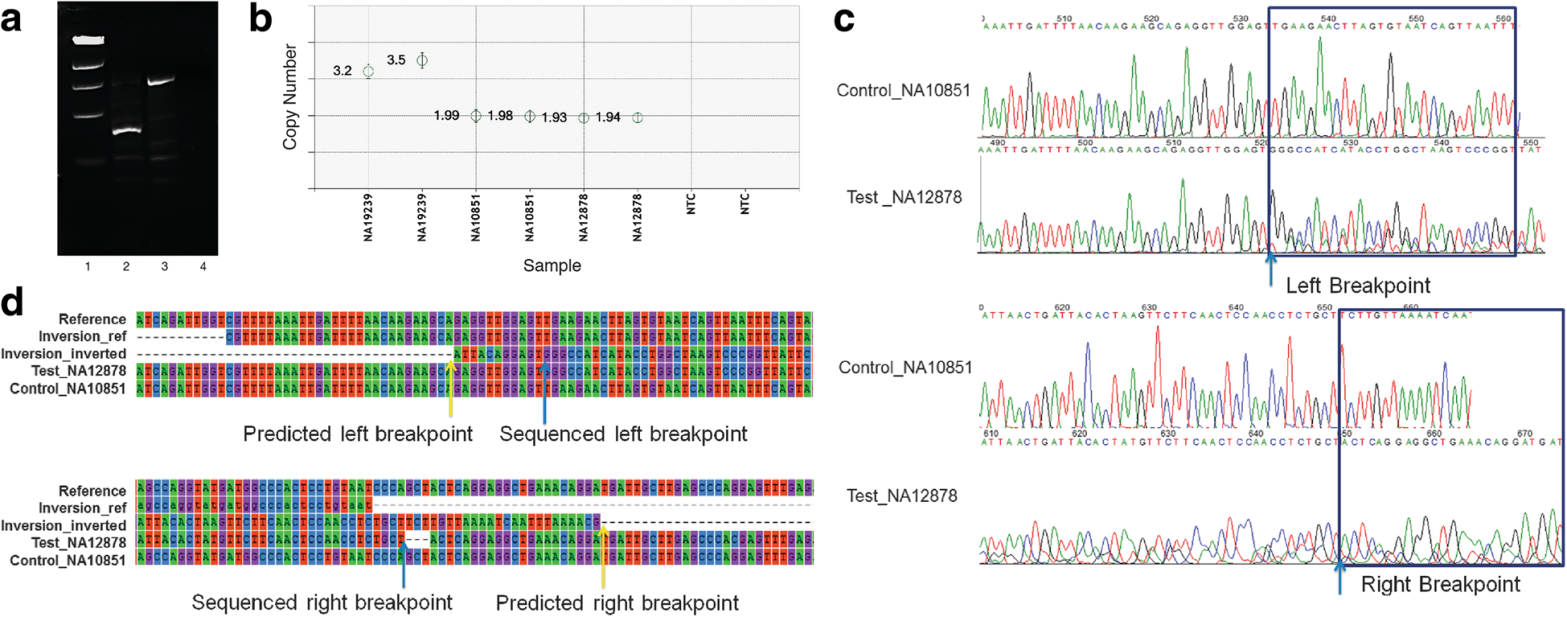
In vitro validation techniques. **a** Example of PCR validation on deletion (Del_218). Lane 1 is the DNA marker; Lane 2 is the test sample; Lane 3 is the reference control; Lane 4 is the no template control (NTC). The test sample has a deletion in the target position which makes its amplification PCR size smaller than reference control. **b** Example of ddPCR validation on duplication (Dup_1158). NA19239 is the test sample. NA10851 and NA12878 are reference controls. NTC is the no template control. Duplicates were run to avoid random experimental error in all ddPCR experiments. NA19239 has an amplification compared to the control. This candidate has been validated. **c** Example of Sanger sequencing validation on Inversion (Inv_190). **d** Sanger sequencing chromatogram to identify inversion. The *arrows* indicate the breakpoints from where the sequences between test sample and control become different with each other. The *yellow arrows* indicated the predicted left and right breakpoints using FusorSV algorithm and the *blue arrows* indicated the sequenced breakpoints by Sanger sequencing. Reference: reference genomic sequences (GRCh37/hg19 Assembly) extracted from UCHC Genome Browser; Inversion_Ref: predicted inversion sequences by FusorSV; Inversion_inverted: inverted inversion sequences; Test_NA12878: nucleotide sequences from Sanger sequencing on test sample NA12878. Control_NA10851: nucleotide sequences from Sanger sequencing on control sample NA10851

The final *FusorSV* callset included 843 SV calls (610 deletions, 202 duplications, and 31 inversions) that were novel and not part of the 1000GP phase 3 release [21] for these 27 samples. We selected a subset from these novel SV calls to perform in vitro validation experiments (Fig. 5). Deletions were validated by polymerase chain reaction (PCR) and droplet digital PCR (ddPCR) [24, 25], using comprehensively studied samples NA12878 and NA10851 as controls (primers can be found in Additional file 4: Table S3). A deletion was considered validated when the test sample amplified a smaller fragment product compared to the control sample in the PCR experiment or the test sample has < 2 copies and the control samples have two copies in ddPCR experiments. In total, we validated 86.1% (31/36) of the randomly selected novel deletions (Table 1 and Additional file 4: Table S3).

**Table 1:** Summary of in vitro validation of novel SV calls

	Validated	Not validated	Min size (bp)	Max size (bp)	Validation rate (%)	Methods
DEL	31	5	401	13,572	86.1	PCR/ddPCR
DUP	27	3	1897	30,370	90.0	ddPCR
INV	7	2	141	1568	77.8	PCR/Sanger sequencing/PacBio

*DEL* deletions, *DUP* duplications, *INV* inversions

To verify inferred duplications, ddPCR technology was used to assess the copy number for the test samples and controls. A duplication was considered validated in a test sample if it had significant amplification compared to both reference controls, NA10851 and NA12878. In this manner, 90% (27/30) randomly selected novel duplications were validated by our customized ddPCR assays (Table 1 and Additional file 4: Table S3).

We applied PCR and Sanger sequencing to verify inversions. In PCR, an inversion was considered as validated if the test sample amplified the inversion allele by primers A/C and B/D and the control sample did not amplify the inversion allele by primers A/C and B/D (Additional file 1: Figure S5). In Sanger sequencing, the sequences of the target region from the test sample were compared to reference sequences to determine whether an inversion candidate was validated or not. For one of the test samples, we had some low coverage whole genome PacBio sequencing data available over the target region. Interrogation of the read alignments across the predicted inversion confirmed the inversion. By the combination of these methods, 77.8% (7/9) of the inversion candidates were validated in this study (Table 1 and Additional file 4: Table S3).

Overall, the experimental validation results further confirmed the increased accuracy of the *FusorSV* method. It is worth noting that all SVs selected for in vitro validation are novel SVs that were not previously reported in the 1000GP callset [21]. Our results indicated a high validation rate for deletion and duplication SVs. However, as observed in previous studies, it remains technically challenging to validate inversions by PCR/Sanger sequencing methods due to the higher complexity at the breakpoints. Indeed, the most recent 1000GP manuscript [1] suggested that only 20% of inversions were actually simple inversions with two clearly defined breakpoints. A majority of inversions were, in fact, inverted duplications or more complex events.

### *FusorSV* implementation and future updates

*SVE* including *FusorSV* is implemented as an extensible and open source algorithm for incorporating the best published SV-calling algorithms such as LUMPY, DELLY, and GenomeSTRiP. As the field evolves and new technologies and higher performing algorithms emerge, *SVE* can be updated to include the latest and most current algorithms. To add a new algorithm, the provided algorithm map file can be updated and subsequent training is performed on a new set of VCF files to recalculate the revised expectation values for each bin. New technologies can be incorporated at the model level, since each model characterizes the limitations of the algorithms with respect to that genomic technology. Model mixture techniques described in the “Methods” section provide a straightforward way to incorporate several models that are each fit to a technology and provide the ability to include many studies together such as Genome in a Bottle Consortium, Simons Genome Diversity Project, and the 1000 Genomes project.

## Discussion

### A novel paradigm for merging SV calls

In this study, we have described *FusorSV*, an extensible computational framework that is able to incorporate an arbitrary number of algorithms under a fusion model. *FusorSV* uses discriminating features to promote subsets of algorithms that are complementary to each other. For example, DELLY and LUMPY appear similar in methodology and produce similar DEL calls but diverge significantly for INV calls. Hence, *FusorSV* empirically determines the similarity/dissimilarity of each combination of SV calling algorithms for each SV type and would select to combine DELLY and LUMPY inversion calls and ignore the combination of their deletion calls. This can be seen in a much more rigorous manner in Additional file 1: Figure S4B, where several algorithm pairs show high scores and produced extremely similar callsets across the 27-sample study. We provide Additional file 5: Table S5, which provides the E-Values for every combination of SV callers across all the bins. The top panel of Additional file 1: Figure S6 shows how the various combinations of the eight algorithms are selected to contribute to the final *FusorSV* output callset. Based on the described training method, we score the expectation value of each possible combination of the input callers, and then use a cutoff to determine which combinations score high enough to contribute information to the output. The lower panel of Additional file 1: Figure S6 shows the top ten combinations of SV callers for deletions, duplications, as well as inversions. The panel also shows which SV size bins do these combinations contribute to (performance plots for all samples are shown in Additional file 1: Figure S7). Integration of individual algorithm performance, along with the pairwise similarity/dissimilarity of algorithms across SV types, allows *FusorSV* to select subsets of algorithms for each SV type that are more comprehensive and balanced (maximum discovery with minimal subset false positives) in nature.

To fully leverage the benefits of *FusorSV*, a sufficient number of diverse algorithms interrogating genomes with coverage > 20X is recommended [7]. In our study, MetaSV was too restrictive in making DEL calls when the VCF filter was employed, which led to a lower recall and fewer total calls being made than most other algorithms (Figs. 3 and 4). The MetaSV result could be improved by using more algorithms in the ensemble, which could then increase the consensus in the variant region but it may also decrease the precision for MetaSV. By using prior knowledge such as the 1000GP Phase 3 SV callset [1, 16], we engaged in an information-based decision process that performed well even with a large and redundant number of SV callsets as input.

### Extensible nature of *FusorSV*

A central goal behind the design of *SVE*/*FusorSV* was to build a computational framework that not only combines the current state of the art SV callers in an ensemble, but also to be extensible so that the framework evolves with the field. New technologies and platform such as Pacific Biosciences Single Molecule, Real-Time (SMRT) sequencing (PacBio) as well as 10X Genomics could be added in the future. However, it is important to provide a truth set of SV calls derived from new technology for retraining the *FusorSV* fusion model. Otherwise application of a fusion model derived from one technology might unduly penalize discovery using another technology. However, this feature ensures that *FusorSV* will perform at least as well as the best existing algorithms/technologies and are included in the framework.

### Duplications, inversions, and other complex SV types

A limitation of any supervised-learning method is having training data representative of information in the test data. However, in our case, SV callsets from cnMOPS, CNVnator, and GenomeSTRiP only reproduce (precision score of < 1%, 22%, and 26%, respectively) of the DUP calls from the 1000GP Phase 3 SV callset [1, 16]. Therefore, *FusorSV* was limited in its ability to call duplications. Since translocation and more complex SV types were not provided in the 1000GP Phase 3 SV callset [1, 16], we did not include these SV types in the 27-sample study. A possible solution to this would be to add a truth set that includes translocations and complex SVs and retrain the model or simulate these types only and use model mixture.

The inherent complexity of inversion events makes it not only difficult to identify them using short read sequencing data but also makes validation efforts problematic, which remains a topic for future improvements in *FusorSV* and for the community. Once we have better algorithms for calling inversions from Illumina short-read sequencing data, these new algorithms can be added to *SVE* and *FusorSV* model, thereby improving our ability to call inversions. Another approach that can be used to detect inversions more accurately is to employ complementary technologies that provide long-range information such as PacBio, 10X genomics, and BioNano Genomics. These can be added as new technologies during the training step in the *FusorSV* framework and once again highlights the most unique and impactful feature of the *FusorSV* framework, extensibility.

## Conclusions

The recent advancements in short-read sequencing technologies (Illumina XTen/NovaSeq – Illumina Inc.) have significantly decreased the costs of WGS and enabled many large-scale consortium projects, such as the Trans-Omics for Precision Medicine Program (TOPMED; https://www.nhlbi.nih.gov/science/trans-omics-precision-medicine-topmed-program) and Genome Sequencing Program (GSP; https://www.genome.gov/10001691/nhgri-genome-sequencing-program-gsp/) as well as clinical centers to include WGS-based SV analysis in their fields. This study highlights how an ensemble of algorithms with a data mining approach to callset merging provides higher quality SV calls than what is possible with a single algorithm. We believe that *FusorSV* will provide a flexible workflow and SV calling framework as well as serve as the gold standard for SV calling for the research community. The SVE and FusovSV are available at https://github.com/TheJacksonLaboratory/SVE.

## Methods

### Calls, callsets, and observations

A call is defined as a tuple that uniquely identifies a change in the reference using reference and test genome coordinates on a flattened reference with the following elements:

*s*_*start*_ - inclusive starting flattened coordinate position of the source

*s*_*end*_ - inclusive ending flattened position of the source

*sv*_*type*_ - simple canonical structural variation type that is one of the following:

INS - a sequence inserted in the sample that is not present in the reference

DEL - a sequence in the reference that is deleted in the sample

DUP - a sequence in the reference that is duplicated in the sample

INV - a sequence in the reference that appears in reverse order in the sample

TRA - a sequence in the reference that appears in a different location in the sample

*d*_*start*_ - inclusive flattened starting position of the destination

*d*_*end*_ - inclusive flattened ending position of the destination

*a*_*seq*_ - alternate sequence for INS types

A callset *c*_*i*_ is then defined as the set of calls from caller *i*.

For *n* callers we then denote observation *y* as: ${S}^y=\left\{{c}_1^y,{c}_2^y,\cdots {c}_n^y\right\}.$

Pooled observations are used to maximize the amount of information used for performance measurement. The use of flattened coordinates provides a simplified analysis, while using both source and destination coordinates allows translocations to be represented.

#### Performance measurement

Event-based scoring mechanisms such as F-measure focus on breakpoints and may fail to capture amounts of matching base pairs between two events. For example, an F1 score could be very low for a 1-Mbp deletion if it was called as three events in one callset and one event in another callset. Alternatively, the Jaccard base-pair similarity metric (J) computes the number of intersecting bases between two callsets. For any two callsets *c*_*i*_ and *c*_*j*_ we define four performance metrics:

Precision or the number of the calls in callset *c*_*i*_ that were overlapped by at least one of the calls in truth set *c*_*j*_:

$$ prec=\mid {c}_i\  overlap\ with\ {c}_j\mid \div \mid {c}_i\mid $$

Recall or the number of calls in the truth set *c*_*j*_ that were overlapped by at least one of the calls in callset *c*_*i*_:

$$ rec=\mid {c}_j\  overlap\ with\ {c}_i\mid \div \mid {c}_j\mid $$

F-measure (F1 score) of precision and recall provides a weighted averaging of both precision and recall, which is a measure of event accuracy:

$$ F1=\frac{2\times prec\times rec}{prec+ rec} $$

Jaccard base-pair similarity or the magnitude of the number of intersecting base pairs in callset *c*_*i*_ and callset *c*_*j*_ divided by the union size: *J* = |*c*_*i*_ ∩ *c*_*j*_| ÷ |*c*_*i*_ ∪ *c*_*j*_|.

A distance is written for each as: *δ*(*c*_*i*_, *c*_*j*_) = 1 − *q*(*c*_*i*_, *c*_*j*_), *q* ∈ {*prec*, *rec*, *F*1, *J*}.

#### Additive group expectation

To assemble the most complementary and highest performing ensemble, we use the maximum possible observations to correct shared caller information within a subset of callers referred to as a group. Two high-performing but dissimilar callers are given an increased value, while high-performing but similar callers are given a decreased value. We estimated the expected value of each group in the powerset by iterative multiplication of elements in the group shown in Eq. 1, using the total ordering of each callset’s performance. This achieves the dual goal of promoting high performance and group dissimilarity (see Fig. 2 and “Methods”).

Let *x* be the number of input observations with *n* callsets each.

Let true callsets for each of the *x* observations be: ${c}_t=\left\{{c}_t^1,{c}_t^2\cdots, {c}_t^x\right\},$

Let the weight for each observation be: *w* = {*w*_1_, *w*_2_,  ⋅ , *w*_*x*_},

Let the pairwise distance matrix be: $D\left({c}_i,{c}_j,w\right)=\frac{\sum_{y=1}^x{w}_y\delta \left({c}_i^y,{c}_j^y\right)}{\sum_{y=1}^x{w}_y},\forall i,j\in \left\{1,2,\cdots, n,t\right\},$

then for every subset in the powerset of callers, we estimate the expectation as:

1 $$ {\displaystyle \begin{array}{l}\forall G\subseteq \mathrm{\mathbb{P}}\left(\left\{1,2,\cdots, n\right\}\right):\\ {}\kern1.56em {G}^{\prime }=\mathrm{the}\kern0.17em \mathrm{lexicographical}\kern0.17em \mathrm{ordering}\kern0.17em \mathrm{of}\;\mathrm{G}\;\mathrm{by}\;\mathrm{the}\kern0.17em \mathrm{distances}:\kern0.36em \\ {}\kern1.56em \left(D\left({c}_i,{c}_t,w\right),1-D\left({c}_i,{c}_j,w\right)\right),\forall i,j\in G,i\ne j\\ {}\kern1.44em E\left({G}^{\prime}\right)=\prod \limits_{i\in {G}^{\hbox{'}}}\left(\left[1-D\left({c}_i,{c}_t,w\right)\right]\;\underset{j\in {G}^{\hbox{'}}\backslash \left\{i\right\}}{\max}\;\left\{D\left({c}_j,{c}_i,w\right)\right\}\;\right)\end{array}} $$

#### Updating additive group expectation

When new observations are available, the prior expectation can be updated by extending the input observations and weights from the *x* in the prior by the *v* in the new:

$$ {S}^1=\left\{{c}_1^1,\cdots, {c}_n^1\right\},\cdots, {S}^{x+v}=\left\{{c}_1^{x+v},\cdots {c}_n^{x+v}\right\}\kern0.24em \mathrm{and}\kern0.24em w=\left\{{w}_1,\cdots, {w}_x,{w}_{x+1},\cdots, {w}_{x+v}\right\} $$

(a) When validation is possible, the prior true dataset is updated by subtracting the calls that failed validation from the prior true and creating new post-validation observations that are constructed by clipping callsets to the validation flanking regions. In this case, we need to redefine the distance matrix and include all true observations and weights:

$$ {c}_t=\left\{{c}_t^1,{c}_t^2,\cdots, {c}_t^x,{c}_t^{x+1},\cdots, {c}_t^{x+v}\right\}, $$

$$ {D}^{\prime}\left({c}_i,{c}_jw\right)=\frac{\sum_{y=1}^{x+v}{w}_y\delta \left({c}_i^y,{c}_j^y\right)}{\sum_{y=1}^{x+v}{w}_u},\forall i,j\in \left\{1,2,\cdots, n,\mathrm{t}\right\} $$

Equation 1 is then used with *D*^′^(*c*_*i,*_*c*_*j*_, *w*) on the updated input to yield a new expectation with the prior and newly incorporated data together into the estimate as:

2 $$ {\displaystyle \begin{array}{l}\forall G\subseteq \mathrm{\mathbb{P}}\left(\left\{1,2,\cdots, n\right\}\right):\\ {}\kern1.44em {G}^{\prime }=\mathrm{the}\kern0.17em \mathrm{lexicographical}\kern0.17em \mathrm{ordering}\kern0.17em \mathrm{of}\;\mathrm{G}\;\mathrm{by}\;\mathrm{the}\kern0.17em \mathrm{distances}:\\ {}\kern1.44em \left({D}^{\prime}\left({c}_i,{c}_t,w\right),1-{D}^{\prime}\left({c}_i,{c}_j,w\right)\right),\forall i,j\in G,i\ne j\\ {}\kern1.44em E\left({G}^{\prime}\right)=\coprod \limits_{i\in {G}^{\hbox{'}}}\left(\left[1-{D}^{\prime}\left({c}_i,{c}_t,w\right)\right]\;\underset{j\in {G}^{\hbox{'}}\left\{i\right\}}{\max}\;\left\{{D}^{\prime}\left({c}_j,{c}_i,w\right)\right\}\right)\end{array}} $$

(b) When true callsets are not available for new observations, the prior truth is used from Eq. 1, while the new observations are mixed into the prior to correct the shared information in each group which yields:

$$ {c}_t=\left\{{c}_t^1,{c}_t^2,\cdots, {c}_t^x,{c}_t^x\right\}, $$

$$ \mathbb{D}\ \left({c}_i,{c}_j,w\right)=\frac{\sum_{y=1}^x{w}_y\delta \left({c}_i^y,{c}_j^y\right)}{\sum_{y=1}^x{w}_y},\forall i,j\in \left\{1,2,\cdots, n,t\right\}, $$

$$ \mathbf{D}\left({c}_i,{c}_j,w\right)=\frac{\sum_{y=1}^{x+v}{w}_y\delta \left({c}_i^y,{c}_j^y\right)}{\sum_{y=1}^{x+v}{w}_y},\forall i,j\in \left\{1,2,\cdots, n\right\}, $$

3 $$ {\displaystyle \begin{array}{l}\forall G\subseteq \mathrm{\mathbb{P}}\left(\left\{1,2,\cdots, n\right\}\right):\\ {}\kern1.56em {G}^{\prime }=\mathrm{the}\kern0.17em \mathrm{lexicographical}\kern0.17em \mathrm{ordering}\kern0.17em \mathrm{of}\;\mathrm{G}\;\mathrm{by}\;\mathrm{the}\kern0.17em \mathrm{distances}:\\ {}\kern1.56em \left(\mathbb{D}\ \left({c}_i,{c}_t,w\right),1-\mathbb{D}\left({c}_i,{c}_j,w\right)\right),\forall i,j\in G,i\ne j\\ {}\kern1.56em E\left({G}^{\prime}\right)=\coprod \limits_{i\in {G}^{\prime }}\left(\left[1-\mathbb{D}\left({c}_i,{c}_t,w\right)\right]\;\underset{j\in {G}^{\prime}\backslash \left\{i\right\}}{\max}\left\{\mathbf{D}\left({c}_j,{c}_i,w\right)\right\}\right)\;\end{array}} $$

#### Optimal call filtering

Using the expectation estimates for each group as a measure of call quality, we project calls for each observation that has a true callset into a single space assigning the expectation value computed in Eq. 1 to the weight in each segment. Determining the value among all the available expectations that maximizes performance across all observations then fits the model. We locate this value exactly by exhaustive search when the number of callsets *n* is small and approximately using expectation maximization (EM) when *n* grows large. This search is computed constructively where a proposal callset *c*_*p*_ is generated by removing and fusing together projected segments below the current search value *p* (see Fig. 2 and “Methods”):

4 $$ \alpha =\underset{p\in \left\{E(G)\right\},\forall G\subseteq \mathrm{\mathbb{P}}\left(\left\{1,\cdots, n\right\}\right)}{\mathrm{argmin}}\left\{D\left({c}_p,{c}_t,w\right)\right\} $$

#### Breakpoint smoothing and priority merging in discovery

Using the training observations:

${S}^1=\left\{{c}_1^1,{c}_2^1,\cdots, {c}_n^1\right\},{S}^2=\left\{{c}_1^2,{c}_2^2,\cdots, {c}_n^2\right\},\cdots, {S}^x=\left\{{c}_1^x,{c}_2^x,\cdots {c}_n^x\right\}$ and true callsets:

$$ {c}_t=\left\{{c}_t^1,{c}_t^2,\cdots, {c}_t^x\right\} $$ with weighting *w* = {*w*_1_, *w*_2_,  ⋅ , *w*_*x*_}.

We construct the empirical left and right breakpoint differential distributions for every caller *c*_*i*_ ∈ {*c*_1_, *c*_2_, ⋯, *c*_*n*_} denoted as *z*_**L,***i*_ and *z*_**R,***i*_ for every call in *c*_*i*_ that overlaps a call in *c*_*t*,_

Let the standard deviation estimate for each distribution be: ${\widehat{\sigma}}_{\mathbf{L},i}$ and ${\widehat{\sigma}}_{\mathbf{R},i}$

Let each sample in discovery be denoted as *S*^*y*^ ∈ {*S*^*x* + 1^, *S*^*x* + 2^, ⋯, *S*^*x* + *v*^} with every resulting fused discovery call that passes the filter as: *f*^*y*, 1^ ∈ {*j*, *f*^*y*, 1^, *f*^*y*, 2^, ⋯, *f*^*y*, *z*^} and contributing left and right breakpoints of the contributing call for caller *i* in *f*^*y*, *j*^ as ${c}_{\mathbf{L},i,j}^y,{c}_{\mathbf{R},i,\mathrm{j}}^y,$

then we smooth the reported breakpoints using the sample standard deviation proportions for each caller as the weighting mechanism:

5 $$ {\hat{f}}_{\mathbf{L}}^{y,j}={f}_{\mathbf{L}}^{y,j}+\sum \limits_{a\in {f}_{\mathbf{L}}^{y,j}}\left(\left[1-{\hat{\sigma}}_{\mathbf{L},a/}\sum \limits_{b\in {f}_{\mathbf{L}}^{y,j}}{\hat{\sigma}}_{\mathbf{L},b}\right]\kern0.28em {c}_{\mathbf{L},a,j}^y\right)\kern0.96em ,{\hat{f}}_{\mathbf{R}}^{y,j}={f}_{\mathbf{R}}^{y,j}+\sum \limits_{a\in {f}_{\mathbf{R}}^{y,j}}\left(\left[1-{\hat{\sigma}}_{\mathbf{R},a/}\sum \limits_{b\in {f}_{\mathbf{R}}^{y,j}}{\hat{\sigma}}_{\mathbf{R},b}\right]\kern0.28em {c}_{\mathbf{R},a,j}^y\right) $$

When each ${\widehat{f}}^{y,j}$ is completed, they are merged back into a single callset using a priority merge step that keeps higher expectation calls.

#### Model selection and diagnostics

Knowledge-based models are dependent on having similar conditions so diagnostics should be completed to ensure a high quality result. Depth of coverage, read length, and sequencing platform are important variables that affect the number and quality of calls characterized during Training. Several models can be constructed before Discovery (using low and high coverage and simulation datasets) and before the application of Eq. 3, a model selection step orders the available models by proximity to the new data. A final diagnostic using a divergence test (Mantel, 1967) provides guidance to proceed with Discovery if the selected or smoothed model is similar to the newly generated data.

### *FusorSV* validation methods

#### Samples

The test samples used for in vitro validation experiments in this study were NA19239, NA19238, NA12878, HG00419, NA18525, NA196525, and NA19017. NA10851 was selected as reference control for all experiments. NA12878 was used as second control if it was not used as a test genome. All samples were obtained from the Coriell Institute for Medical Research.

#### Primer and probe design

##### Deletions

To design PCR primers for deletion validation, the following pipeline was applied. First, the genomic sequence of a 500-bp region next to each SV breakpoint was extracted from the UCSC Genome Browser on GRCh37/hg19 Assembly. Second, Primer3 Plus [26] was used to compute a set of primer pairs flanking the breakpoint for these regions. 200 bp from each side of breakpoint was excluded from the primer design to avoid the potential uncertainty of breakpoints. Third, the quality score of the primers was checked using Netprimer (PREMIER Biosoft International, Palo Alto, CA, USA) software. The primer would not be used if the quality score was < 80. Fourth, all primer pairs were tested for their uniqueness across the human genome using In Silico PCR from the UCSC Genome Browser. BLAT [27] search was also performed at the same time to make sure all primer candidates have only one hit in the human genome. Finally, the NCBI 1000 Genomes Browser was used to check whether there were any SNPs in the primer or probe binding region. When this process did not result in a valid primer pair, the size of the regions for which primers were designed was increased from 500 bp to 750 bp and the search for valid primers was repeated.

##### Duplications

For each selected duplication candidate, 1000 bp of genomic sequence in the middle of the duplication region were selected to design customized ddPCR assays. The ddPCR primers were designed following same procedure as PCR primers except for a few differences. The ddPCR product size was limited to 60–100 bp to ensure high efficiency. For probe design, any probe with the nucleotide G at its 5′ end was excluded because this may quench the fluorescence signal after hydrolysis. In addition, the T_m_ value of the probe was set 3–10 °C higher than that of the corresponding primers. If this does not result in a valid primer/probe set, another 1000 bp next to the previously selected genomic region were selected and the process repeated.

##### Inversions

A set of four primers was designed for each selected inversion candidate. Forward primer A and reverse primer D were designed to be “outside primers,” flanking the predicted inversion breakpoints, and reverse primer B and forward primer C were designed to be “inside primers” for the predicted inversion (Additional file 1: Figure S5). PCRs were performed for primer combinations A/B, C/D, A/C, and B/D. The reference allele was amplified using primer combinations A/B and C/D, whereas the inversion allele was amplified using primer combinations A/C and B/D.

##### PCR

PCR amplifications were performed in 25-μL reactions using the BioRad DNA Engine Peltier Thermal Cycler and BioRad c1000 Touch Thermal Cycler. Each PCR reaction contained 10 ng of template DNA; 1X PCR buffer (50 mM KCl; 10 mM Tris-HCl, pH 8.3); 0.2 mM dNTPs; 250 nM of each primer; 1.5 mM MgCl_2_; and 1 U Platinum Taq DNA polymerase. PCR reactions were performed under the following conditions: initial denaturation at 94 °C for 2 min, followed by 35 cycles of denaturation at 94 °C for 30 s, annealing at 52–58 °C (depending on the Tm value of primers), and extension at 72 °C for 30–120 s (depending on the predicted PCR amplicon size), and a final extension at 72 °C for 10 min. Aliquots of 5 μL of PCR products were electrophoresed in 2% agarose gels (1× TAE) containing 0.1 μg/mL SYBR Gold (Molecular Probes Inc.) for 45–120 min at 100–120 V. DNA fragments were visualized with UV and images were saved using a G:BOX Chemi systems (Syngene USA) instrument.

### Droplet digital PCR (ddPCR)

The ddPCR reactions were performed following the Bio-Rad QX200™ system manufacturer protocol. Briefly, 10 ng DNA template was mixed with a 2× ddPCR Mastermix, *Hind*III enzyme, 20× primer, and both FAM and HEX-labeled probes) to a final volume of 20 μL. Each reaction mixture was then loaded into the sample well of an eight-channel droplet generator cartridge. A volume of 60 μL of droplet generation oil was loaded into the oil well for each channel and covered with a gasket. The cartridge was placed into the Bio-Rad QX200™ Droplet Generator. After the droplets were generated in the droplet well, they were transferred into a 96-well PCR plate and then heat-sealed with a foil seal. PCR amplification was performed using a C1000 Touch thermal cycler and, once completed, the 96-well PCR plate was loaded on the QX200™ Droplet Reader. All experiments had two normal controls (NA12878 and NA10851) or one normal control NA10851, if NA12878 was the test sample, and a no-template control (NTC) with water. All samples and controls were run in duplicate and data from any well with < 8000 droplets were treated as failed QC and excluded for downstream analysis. The ddPCR data were analyzed with QuantaSoft™ software.

### Sanger sequencing

The sequencing PCR was performed in a 50-uL reaction and the PCR product was run in a 1% Agarose gel with TAE to separate the DNA fragments. The target band was cut from the gel and purified using the Gel Extraction and PCR Clean-Up Kit (Clontech Laboratories). The DNA concentrations were measured using the NanoDrop™ 2000 instrument. The DNA concentration for Sanger sequencing was adjusted to be in the range of 10–20 ng/μL. Samples were then sent off to Eton Bioscience (Charlestown, MA, USA) to be sequenced. The MEGA program [28] was used for the analysis of the Sanger sequencing data.

## Additional files

Additional file 1: This file contains **Figures S1**–**S7.** (DOCX 5111 kb)

Additional file 2: Shell script used for population SV calling using GenomeSTRiP. (SH 10 kb)

Additional file 3: This file contains the supplemental methods. (DOCX 163 kb)

Additional file 4: This file contains **Tables S1**–**S4.** (DOCX 67 kb)

Additional file 5: This file contains **Table S5.** (XLSX 145 kb)

Additional file 6: Reviewer reports and Author’s response to reviewers. (DOCX 34 kb)

## Footnotes

1 Compatible with SVE but not included.
