# Supplementary material for: FusorSV: an algorithm for optimally combining data from multiple structural variation detection methods
Source: Genome Biol. 2018 Mar 20;19:38. doi: 10.1186/s13059-018-1404-6 (PMC5859555; doi:10.1186/s13059-018-1404-6)
Supplement: Supplementary file 1 — This file contains Figures S1–S7. (DOCX 5111 kb) [file 13059_2018_1404_MOESM1_ESM.docx]

**Additional file**

***
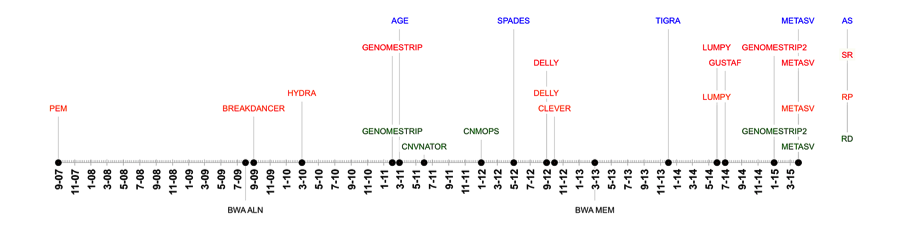
Additional file 1: Figure S1. Algorithm Timeline Figure*: RD –** denotes read depth distribution based methods, **RP –** denotes read pair distribution based methods, **SR** – Split read information based methods, **AS** – Assembly information based methods. *From early work with paired-end sequencing (****PEM****), advancements were first made in alignment via the* ***bwa aln*** *short read aligner followed closely by two read pair distributions methods* ***BreakDancer*** *and* ***Hydra****. The next major development was in the use of read depth strategies where GC content normalization, sequence length normalizations and region masking was used by* ***GenomeSTRiP*** *providing long SV range calling that was not previously available. Assembly methods were developed and advanced from* ***AGE*** *to* ***SPAdes*** *and* ***TIGRA****. This was followed by* ***DELLY*** *that utilizes RP and SR strategies. A major upgrade to sequence alignment mapping was made next:* ***bwa mem****, which has become the most widely used algorithm for sequence alignment. The timeline ends in a strong trend for multi-strategy methods like* ***GenomeSTRiP2****,* ***LUMPY*** *and* ***MetaSV*** *that provide higher accuracy than preceding algorithms in the timeline.*

***
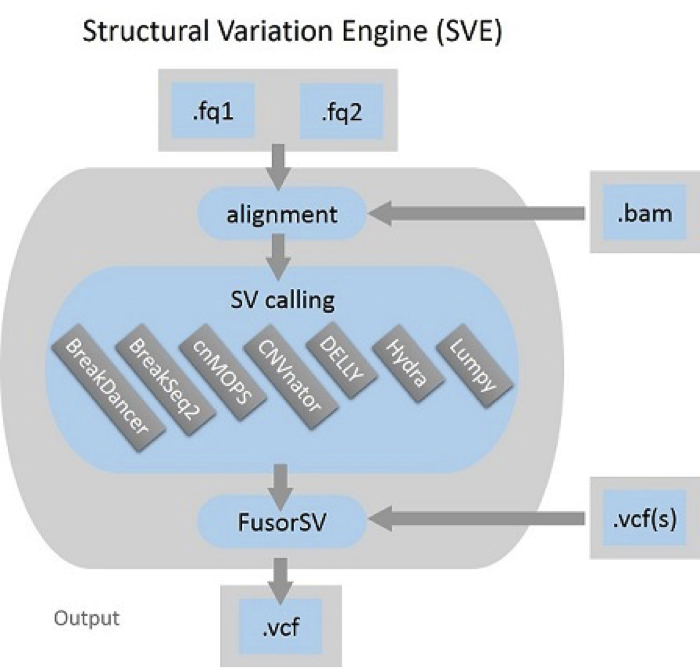
***

***Additional file 1: Figure S2: Sequence Analysis Pipeline Overview.*** *FASTQ files are first aligned to a FASTA reference genome in the* ***alignment*** *step. Next,* ***SV Calling*** *is performed for each calling algorithm and then output to the* ***FusorSV*** *step, where FusorSV is used to make a single VCF file from the input given prior knowledge obtained for each caller and the caller to caller interactions as detailed in methods and materials. SVE can be applied by given FASTQs, BAMs or VCFs, and generate a unified VCF. Each SV-calling algorithm in SVE has a unique ID; BreakDancer: 4, BreakSeq2: 35, cnMOPS: 9, CNVnator: 10, DELLY: 11, GenomeSTRiP: 14 (not embedded in SVE due to the license but FusorSV is able to handle the output from it) and LUMPY: 18 (Supplemental Table 4).*

***
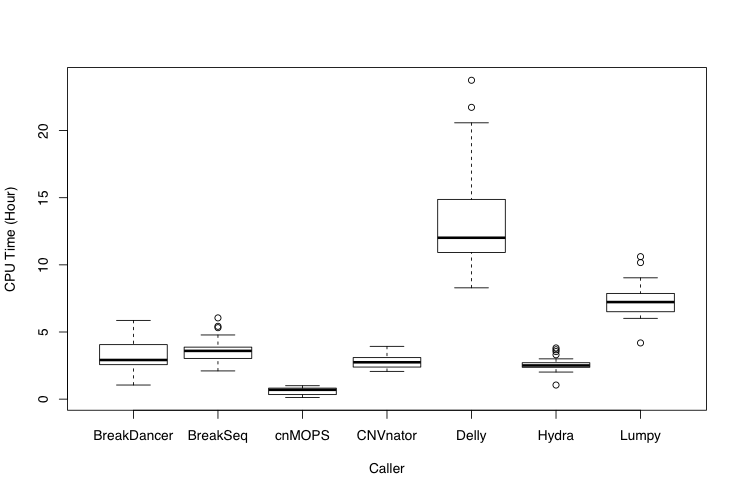
***

***Additional file 1: Figure S3: Running time.*** *CPU time for seven SV callers running by SVE on the 27 High-coverage (50X) 1000GP samples. GenomeSTRiP is not performed via SVE due to the license and high computational resource it requires and it was done by using a cluster. DELLY took a long running time because we sequentially run DELLY for five SV types, del, dup, inv, tra and ins.*

***
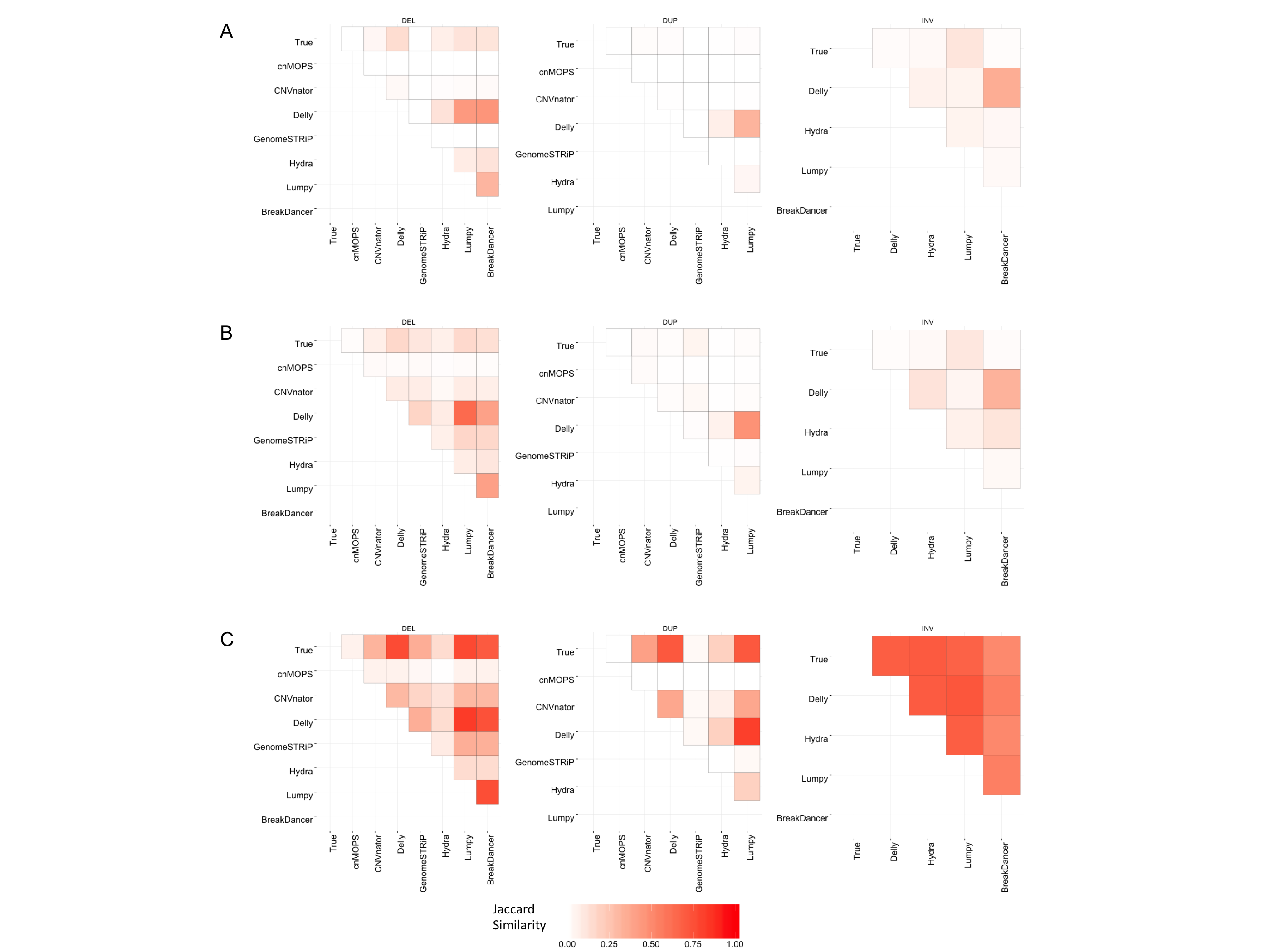
Additional file 1: Figure S4. Algorithm-to-Algorithm Similarity.*** *Three pairwise studies were conducted with the FusorSV framework with the same algorithms to determine the algorithm-to-algorithm pairwise similarity metrics. (A) 100 Low-coverage (5-10X) 1000GP samples, (B) 27 High-coverage (50X) 1000GP samples, (C) 30 High-coverage (50X) VarSim simulated samples The Truth callset in the top row for each panel depicts the callset used for training.*

***
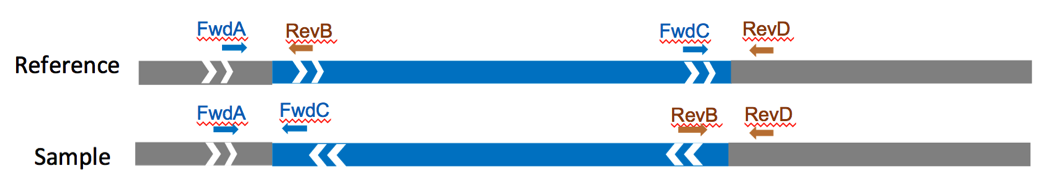
Supplemental Figure S5: PCR based Inversion validation strategy.*** *Cartoon depicting where the Primers were placed for the PCR based Inversion validation experiments. (Fwd: forward, Rev: reverse)*

***Additional file 1: Figure S6: SV caller combinations in the fusorSV fusion model.*** *Top panel shows the 8 SV callers in the ensemble, and how the various combinations (n=2^8^ -1=255) are scored (E-values) in the training model. There are six heatmaps plotted, two each for DELETION/DUPLICATION and INVERSION. The heatmaps on the left are after training, and the heatmaps on the right are after application of the cutoff filter. Each row in the heatmap corresponds to one of the 255 possible combinations of the 8 SV callers and the different columns correspond to the different SV size bins. We plot the calculated expectation value (E-value) for each combination/size bin. All combinations that pass the alpha value cutoff contribute to the FusorSV output call set. Bottom panel show the highest performing combinations and the various bins that they contribute to. For example, for DELETIONS, the highest performing caller combination includes BreakDancer / BreakSeq / CNVnator / Delly / Lumpy and this combination clears the alpha value cutoff for all the different DELETION size bins.*

***Additional file 1: Figure S7: Caller performance by sample.*** *The figure includes three plots per sample, that show the performance (precision vs recall) of the individual callers, including FusorSV separated by Deletions/Duplications and Inversions*
