# Supplementary material for: FusorSV: an algorithm for optimally combining data from multiple structural variation detection methods
Source: Genome Biol. 2018 Mar 20;19:38. doi: 10.1186/s13059-018-1404-6 (PMC5859555; doi:10.1186/s13059-018-1404-6)
Supplement: Supplementary file 3 — This file contains the supplemental methods. (DOCX 163 kb) [file 13059_2018_1404_MOESM3_ESM.docx]

**SUPPLEMENTAL METHODS**

**Read depth (RD)** methods which can make DEL or DUP calls look across the coordinate space of the reference genome and count the number of aligned reads that lie within a preselected analysis window. These counts can be done for properly paired reads, for each pair, or for all in total and any combination therein. These counts are then normalized for GC content and each segment for each sample can be used to infer using an underlying model the loss or gain of copy number. One limitation of RD methods is the resolution, which is governed by the depth of sequencing used and the related window size where high coverage provides the ability to use smaller analysis windows and thus provide better breakpoint accuracy and more certainty of the event’s existence in relation to the reference and sample inputs.

**Read pair (RP)** methods calculate for each read group in each sample the statistics for the insert size between primary alignments as well as the strand and location information to infer INS, DEL, DUP, INV and TRA calls. This mean insert size and standard deviation are typically used to determine when an observed pair of reads has an abnormal insert size which indicates DEL and INS signals depending on where the insert is more or less than the standard deviation cutoff point. The strand information and the alignment of the match pair allow the RP method the ability to make TRA and INV calls. Limitations of the RP method include difficulty with repetitive regions and limited resolution for breakpoints based on the read depth, read length and stability of the insert size for non-variant regions.

**Split read (SR)** methods allow the use of part of reads that map to a section where anchor points are sought on a variant breakpoint where a contiguous section of a read has a high mapping quality but then one end does not. Limitations of Split read methods are the read depth and read length where if more reads are used the chance of having a read land on a variant breakpoint exactly will increase.

**Assembly (AS)** methods can either be guided by the reference or conducted completely de novo where individual reads (and their corresponding mates) are joined together based on inter read sequence overlap to build a contiguous sequence or contig of the total altered sequence. Once assembles are constructed a separate inference step must be used to check that the contig is a realistic representation of the reads that were provided and can be associated to an appropriate variant type. Assembled contigs are then mapped back to the reference to infer the simplest variant type that can explain the mapped assembly configuration. With low read depth sequencing these methods might not have enough reads to successfully anchor to variant breakpoints or to finish longer alternate contigs that span larger variants. Likewise if given a large pool of unmapped reads these methods may not be able to explore all the possible ways to assemble the data in a computationally feasible time frame which often leads to limiting the assembly search.

**The 1000 Genomes Project Phase 3 SV callset used as the truth set.**

<ftp://ftp.ncbi.nlm.nih.gov/pub/dbVar/data/Homo_sapiens/by_study/estd214_1000_Genomes_Consortium_Phase_3/vcf/estd214_1000_Genomes_Consortium_Phase_3.GRCh37.submitted.variant_call.germline.vcf.gz>

**Caller Versions, Command Lines, Supporting Files (references and masks)**

**Reference**

FASTA format with appropriate indexes:

ftp://ftp.1000genomes.ebi.ac.uk/vol1/ftp/technical/reference/human_g1k_v37.fasta.gz

**BreakDancer**

Version: breakdancer-1.4.5

Commands:

perl breakdancer-1.4.5/perl/bam2cfg.pl $SAMPLE.bam –q 30 –n 10000 \

> $SAMPLE.cfg

breakdancer-1.4.5/build/bin/breakdancer-max cfg \

> $SAMPLE.calls

# python

# import utils.breakdancer2vcf as bd

# $SAMPLE.table = bd.read_breakdancer($SAMPLE.calls)

# bd.write_vcf($SAMPLE_S4.vcf, bd.vcf_header($REF),bd.build_vcf($SAMPLE.table))

**BreakSeq**

Version: Breakseq2-2.2

Command:

breakseq2-2.2/scripts/run_breakseq2.py \

--bwa $PATH_TO_BWA \

--samtools $PATH_TO_SAMTOOLS \

--reference $REF \

--bplib_gff *SVE*/data/breakseq_bplib/breakseq2_bplib_20150129.gff \

--work $WORK_DIR \

--bams $BAM \

--nthreads $NUMBER_THREADS \

--min_span 2 \

--window 500 \

--min_overlap 2 \

--junction_length 1000

**cnMOPS**

Version: cn.mops_1.22.0, R-3.3.3, Rsamtools_1.28

Command:

R CMD BATCH *SVE*/src/R-package/installPackage.r logInstall

R-3.3.3/bin/Rscript *SVE*/stages/utils/cnmops.R\

ref_seq=$REF \

in_bams=$BAM \

out_vcf=$SAMPLE_S9.vcf \

upper=0.5 \

lower=-0.9 \

min_seg=2 \

normal=3 \

prior=0.0 \

window=1000 \

min_cnt=0 \

mode=3 \

cir_seg=False \

cores=$NUMBER_THREADS \

cutoff=0.1 \

paired=True

Notes: Automation scripts include a command parser and an automated version of cnMOPS that converts output to VCF file.

**CNVnator**

Version: CNVnator v0.3.3

Commands:

cnvnator_0.3.3/cnvnator \

–unique \

-root $SAMPLE.tree.root \

-tree $SAMPLE.bam

cnvnator_0.3.3/cnvnator \

-root $SAMPLE.tree.root \

-outroot $SAMPLE.root.his.root \

--his 150 \

-d $WORK_DIR

cnvnator_0.3.3/cnvnator \

-root $SAMPLE.root.his.root \

-stat 150

cnvnator_0.3.3/cnvnator \

-root $SAMPLE.root.his.root \

-partition 150

cnvnator_0.3.3/cnvnator \

-root $SAMPLE.root.his.root \

-call 150 \

> $SAMPLE.call

perl *SVE*/src/CNVnator_v0.3.3/cnvnator2VCF.pl \

$SAMPLE.call \

> $SAMPLE_S10.vcf

**DELLY**

Version: delly-0.7.7, bcftools-1.4-22-g68898af

Commands:

delly-0.7.7/src/delly call \

-g $REF \

-n \

-x delly/excludeTemplates/human.hg19.excl.tsv \

-t DEL\

-o $WORK_DIR.DEL.bcf \

$BAM

delly-0.7.7/src/delly call \

-g $REF \

-n \

-x delly/excludeTemplates/human.hg19.excl.tsv \

-t DUP

-o $WORK_DIR.DUP.bcf \

$BAM

delly-0.7.7/src/delly call \

-g $REF \

-n \

-x delly/excludeTemplates/human.hg19.excl.tsv \

-t INV\

-o $WORK_DIR.INV.bcf \

$BAM

delly-0.7.7/src/delly call \

-g $REF \

-n \

-x delly/excludeTemplates/human.hg19.excl.tsv \

-t BND\

-o $WORK_DIR.BND.bcf \

$BAM

delly-0.7.7/src/delly call \

-g $REF \

-n \

-x delly/excludeTemplates/human.hg19.excl.tsv \

-t INS\

-o $WORK_DIR.INS.bcf \

$BAM

bcftools/bcftools concat –a –o $SAMPLE_S11.vcf \

$WORK_DIR.DEL.bcf \

$WORK_DIR.DUP.bcf \

$WORK_DIR.INV.bcf \

$WORK_DIR.BND.bcf \

$WORK_DIR.INS.bcf

**GenomeSTRiP**

Version: svtoolkit_2.00.1665: released May 17, 2016

(Because of license issues we did not include GenomeSTRiP in the SVE. We provide the basic shell script that used to derive the VCFs.

Supporting Files:

(1) official meta data bundle for hg19 based reference at:

ftp://ftp.broadinstitute.org/pub/svtoolkit/reference_metadata_bundles/1000G_phase3_12May2015.tar.gz

(2) DEL and DUP genotyped to individual sample scripts from the *SVE* at:

https://github.com/timothyjamesbecker/*SVE*/blob/master/stages/utils/gs_split_merge.py

Commands:

Notes: DEL calls were derived from the SVDiscovery deletion calling pipeline and the DUP calls were derived from the CNVDiscovery pipeline which were inferred with DEL as integer copy number <= 1.0 and DUP as >= 3.0 (defaults in the script).

#!/bin/bash

#PBS -N GenomeSTRiP_population_g1k # job name

#PBS -S /bin/bash

#PBS -k o # keep job output

#PBS -m abe # send email if job is (a) aborted, when it (b) begins, and when it (e) ends

#PBS -l nodes=1:ppn=6,walltime=344:00:00 # 5 nodes, 14 processor per node, and 22 hours wall-clock time

#PBS -j oe # join standard output and standard error

#PBS -q test # use queue 'test' queue, which has a 344 hour retention set

# Note:

# $1 - file_id # pop

# $2 - file_name # pop.list

# $3 - data_dir, directory containing subject data

# $4 - ref_dir, directory containing reference data

# $5 - top_container, root folder containing all subfolders

# ---------------- Start Time -----------------

echo "START: " `date`

echo ""

# ------------- PATH Executables --------------

source ~/.bashrc # JPL

which java > /dev/null || exit 1

which Rscript > /dev/null || exit 1

which samtools > /dev/null || exit 1

# ------------- Executables Version -----------

echo `java -version`

echo `Rscript --version`

echo `samtools --version`

echo ""

# ------------- Containers / Directory Structure -------------

top_container=GenomeSTRiP_population # pop

file_id=population_g1k # $1

runDir=$top_container/$file_id

# ------------- Sample Data -------------

data_dir=varsim_bams

file_name=population_g1k.list # $2

bam=$data_dir/$file_name

genderMapFile=$data_dir/genderMap.txt

# ------------- Reference Data -------------

refDir=reference_genomes/1000G_phase3

reference_genome=$refDir/human_g1k_hs37d5.fasta

ploidyMapFile=$refDir/human_g1k_hs37d5.ploidymap.txt

genomeMaskFile=$refDir/human_g1k_hs37d5.svmask.fasta

copyNumberMaskFile=$refDir/human_g1k_hs37d5.gcmask.fasta

# ------------- Output Data -------------

sites=$runDir/$file_id.discovery.vcf

genotypes=$runDir/$file_id.genotypes.vcf

# ------------- Executables -------------

# SV_DIR is the SVToolkit installation directory - it must be an exported environment variable.

# SV_TMPDIR is a directory for writing temp files, which may be large if you have a large data set.

#JPL export SV_DIR=`cd .. && pwd`

###export SV_DIR=$HOME/builds/svtoolkit # NB: must be an ENV variable (i.e., export) JPL

SV_TMPDIR=$runDir/tmpdir

echo $SV_DIR # JPL

echo $SV_TMPDIR # JPL

###module load java/1.7.0 # JPL

# ------------- Library & Class PATH -------------

# For SVAltAlign, you must use the version of bwa compatible with Genome STRiP.

###export PATH=${SV_DIR}/bwa:${PATH}

###export LD_LIBRARY_PATH=${SV_DIR}/bwa:${LD_LIBRARY_PATH}

###export LD_LIBRARY_PATH=${DRMAA_LIBRARY_PATH}:${LD_LIBRARY_PATH}

mx="-Xmx4g"

classpath="${SV_DIR}/lib/SVToolkit.jar:${SV_DIR}/lib/gatk/GenomeAnalysisTK.jar:${SV_DIR}/lib/gatk/Queue.jar"

mkdir -p ${runDir}/logs || exit 1

mkdir -p ${runDir}/metadata || exit 1

cd $runDir # $SV_DIR/installtest # JPL

echo "pwd=" `pwd` # JPL

# Display version information.

java -cp ${classpath} ${mx} -jar ${SV_DIR}/lib/SVToolkit.jar

# +--------------------------------------------------------------------+

# | Note: |

# | { Tell Queue kind of jobRunner (e.g., PBS) and its arguments. } |

# | |

# | -gatkJobRunner PbsEngine \ |

# | -jobRunner PbsEngine \ |

# | |

# | -jobNative "-v SV_DIR=$SV_DIR" \ |

# | -jobNative "-v SV_TMPDIR=$SV_TMPDIR" \ |

# | -jobNative "-v PATH=$PATH" \ |

# | -jobNative "-v LD_LIBRARY_PATH=$LD_LIBRARY_PATH" \ |

# | -jobNative "-v classpath=$classpath" \ |

# | -jobNative "-l nodes=1:ppn=6,walltime=344:00:00" \ |

# | -jobNative "-q test" \ |

# | |

# | NOTE: Could all "-v ..." be replaced by single "-S /bin/bash"? |

# | Yes, except SV_TMPDIR and classpath, which are defined |

# | within this script. |

# +--------------------------------------------------------------------+

# ----------------------------------------------------------------------------------------

# --SVPreprocess

# Preprocess a set of input BAM files to generate genome-wide metadata used by other

# Genome STRiP modules. This is a pre-requisite for all other Genome STRiP pipelines.

echo " ================== Preprocessing (`date`) =================="

# Run preprocessing.

# For large scale use, you should use -reduceInsertSizeDistributions, but this is too slow for the installation test.

# The method employed by -computeGCProfiles requires a GC mask and is currently only supported for human genomes.

java -cp ${classpath} ${mx} \

org.broadinstitute.gatk.queue.QCommandLine \

-S ${SV_DIR}/qscript/SVPreprocess.q \

-S ${SV_DIR}/qscript/SVQScript.q \

-gatk ${SV_DIR}/lib/gatk/GenomeAnalysisTK.jar \

-gatkJobRunner PbsEngine \

-jobRunner PbsEngine \

--disableJobReport \

-cp ${classpath} \

-configFile ${SV_DIR}/conf/genstrip_parameters.txt \

-tempDir ${SV_TMPDIR} \

-R ${reference_genome} \

-genomeMaskFile ${genomeMaskFile} \

-copyNumberMaskFile ${copyNumberMaskFile} \

-genderMapFile ${genderMapFile} \

-runDirectory ${runDir} \

-md ${runDir}/metadata \

-disableGATKTraversal \

-useMultiStep \

-reduceInsertSizeDistributions true \

-bamFilesAreDisjoint true \

-computeGCProfiles true \

-computeReadCounts true \

-jobLogDir ${runDir}/logs \

-I ${bam} \

-jobNative "-v SV_DIR=$SV_DIR" \

-jobNative "-v SV_TMPDIR=$SV_TMPDIR" \

-jobNative "-v PATH=$PATH" \

-jobNative "-v LD_LIBRARY_PATH=$LD_LIBRARY_PATH" \

-jobNative "-v classpath=$classpath" \

-jobNative "-l nodes=1:ppn=6,walltime=344:00:00" \

-jobNative "-q test" \

-run \

|| exit 1

# ----------------------------------------------------------------------------------------

# --SVDiscovery

# Run deletion discovery on a set of input BAM files, producing a VCF file of potentially variant sites.

echo " ================== Discovery (`date`) ======================"

# Run discovery.

java -cp ${classpath} ${mx} \

org.broadinstitute.gatk.queue.QCommandLine \

-S ${SV_DIR}/qscript/SVDiscovery.q \

-S ${SV_DIR}/qscript/SVQScript.q \

-gatk ${SV_DIR}/lib/gatk/GenomeAnalysisTK.jar \

-gatkJobRunner PbsEngine \

-jobRunner PbsEngine \

--disableJobReport \

-cp ${classpath} \

-configFile ${SV_DIR}/conf/genstrip_parameters.txt \

-tempDir ${SV_TMPDIR} \

-R ${reference_genome} \

-genomeMaskFile ${genomeMaskFile} \

-genderMapFile ${genderMapFile} \

-runDirectory ${runDir} \

-md ${runDir}/metadata \

-disableGATKTraversal \

-jobLogDir ${runDir}/logs \

-minimumSize 100 \

-maximumSize 1000000 \

-suppressVCFCommandLines \

-P select.validateReadPairs:false \

-I ${bam} \

-O ${sites} \

-jobNative "-v SV_DIR=$SV_DIR" \

-jobNative "-v SV_TMPDIR=$SV_TMPDIR" \

-jobNative "-v PATH=$PATH" \

-jobNative "-v LD_LIBRARY_PATH=$LD_LIBRARY_PATH" \

-jobNative "-v classpath=$classpath" \

-jobNative "-l nodes=1:ppn=6,walltime=344:00:00" \

-jobNative "-q test" \

-run \

|| exit 1

# ----------------------------------------------------------------------------------------

# --SVGenotyper

# Genotype a set of polymorphic structural variation loci described in an input VCF file.

#

# SVDiscovery *VS* SVGenotyper

# Discovery - list of potentially variant sites.

# Genotyping - the process of determining which genetic variants an individual possesses.

echo " ================== Genotyper (`date`) ======================"

# Run genotyping on the discovered sites.

java -cp ${classpath} ${mx} \

org.broadinstitute.gatk.queue.QCommandLine \

-S ${SV_DIR}/qscript/SVGenotyper.q \

-S ${SV_DIR}/qscript/SVQScript.q \

-gatk ${SV_DIR}/lib/gatk/GenomeAnalysisTK.jar \

-gatkJobRunner PbsEngine \

-jobRunner PbsEngine \

--disableJobReport \

-cp ${classpath} \

-configFile ${SV_DIR}/conf/genstrip_parameters.txt \

-tempDir ${SV_TMPDIR} \

-R ${reference_genome} \

-genomeMaskFile ${genomeMaskFile} \

-genderMapFile ${genderMapFile} \

-runDirectory ${runDir} \

-md ${runDir}/metadata \

-disableGATKTraversal \

-jobLogDir ${runDir}/logs \

-I ${bam} \

-vcf ${sites} \

-O ${genotypes} \

-jobNative "-v SV_DIR=$SV_DIR" \

-jobNative "-v SV_TMPDIR=$SV_TMPDIR" \

-jobNative "-v PATH=$PATH" \

-jobNative "-v LD_LIBRARY_PATH=$LD_LIBRARY_PATH" \

-jobNative "-v classpath=$classpath" \

-jobNative "-l nodes=1:ppn=6,walltime=344:00:00" \

-jobNative "-q test" \

-run \

|| exit 1

# ----------------------------------------------------------------------------------------

# --CNVDiscovery

# Run the Genome STRiP 2.0 pipeline for discovery and genotyping of CNVs (including

# deletions, duplications and mCNVs), seeding on read depth.

echo " ================== CNV Discovery (`date`) ======================"

# Run CNV discovery pipline.

java -showversion ${mx} -cp ${classpath} \

org.broadinstitute.gatk.queue.QCommandLine \

-S ${SV_DIR}/qscript/discovery/cnv/CNVDiscoveryPipeline.q \

-S ${SV_DIR}/qscript/SVQScript.q \

-cp ${classpath} \

-gatk ${SV_DIR}/lib/gatk/GenomeAnalysisTK.jar \

-gatkJobRunner PbsEngine \

-jobRunner PbsEngine \

-configFile ${SV_DIR}/conf/genstrip_parameters.txt \

-R ${reference_genome} \

-I ${bam} \

-genderMapFile ${genderMapFile} \

-md ${runDir}/metadata \

-runDirectory ${runDir} \

-jobLogDir ${runDir}/logs \

-tilingWindowSize 5000 \

-tilingWindowOverlap 2500 \

-maximumReferenceGapLength 25000 \

-boundaryPrecision 200 \

-minimumRefinedLength 2500 \

-jobNative "-v SV_DIR=$SV_DIR" \

-jobNative "-v SV_TMPDIR=$SV_TMPDIR" \

-jobNative "-v PATH=$PATH" \

-jobNative "-v LD_LIBRARY_PATH=$LD_LIBRARY_PATH" \

-jobNative "-v classpath=$classpath" \

-jobNative "-l nodes=1:ppn=6,walltime=344:00:00" \

-jobNative "-q test" \

-run \

|| exit 1

Hydra

Version: hydra 0.5.5

Supporting Files: VCF conversion script written by Brad Chapman at:

<https://github.com/TheJacksonLaboratory/SVE/blob/master/stages/utils/hydra_to_vcf.py>

Commands:

bin/sve call -r <FASTA> -g hg19 –a hydra <BAM>

Notes: Reran several times with some adjustments made to the VCF conversion script.

**Lumpy**

Version: lumpy 0.2.13

Command:

bin/sve call -r <FASTA> -g hg19 -a lumpy <BAM>
