# Supplementary material for: FusorSV: an algorithm for optimally combining data from multiple structural variation detection methods
Source: Genome Biol. 2018 Mar 20;19:38. doi: 10.1186/s13059-018-1404-6 (PMC5859555; doi:10.1186/s13059-018-1404-6)
Supplement: Supplementary file 4 — This file contains Tables S1–S4. (DOCX 67 kb) [file 13059_2018_1404_MOESM4_ESM.docx]

**Additional file 4**

| **Caller** | **SV Type** | **prec** | **rec** | **f1** | **J** |
| --- | --- | --- | --- | --- | --- |
| BreakSeq | DEL | 0.56 | 0.21 | 0.31 | 0.19 |
| cnMOPS | DEL | 0.18 | 0.01 | 0.01 | 0.05 |
|  | DUP | 0.04 | 0.01 | 0.02 | 0.09 |
| CNVnator | DEL | 0.44 | 0.16 | 0.24 | 0.10 |
|  | DUP | 0.25 | 0.03 | 0.06 | 0.14 |
| DELLY | DEL | 0.62 | 0.47 | 0.54 | 0.20 |
|  | DUP | 0.05 | 0.01 | 0.01 | 0.03 |
|  | INV | 0.78 | 0.05 | 0.09 | 0.00 |
| GenomeSTRiP | DEL | 0.39 | 0.52 | 0.45 | 0.29 |
|  | DUP | 0.26 | 0.15 | 0.19 | 0.14 |
| Hydra | DEL | 0.42 | 0.46 | 0.44 | 0.21 |
|  | DUP | 0.02 | 0.01 | 0.01 | 0.00 |
|  | INV | 0.55 | 0.10 | 0.16 | 0.00 |
| LUMPY | DEL | 0.59 | 0.48 | 0.53 | 0.21 |
|  | DUP | 0.05 | 0.03 | 0.03 | 0.03 |
|  | INV | 0.60 | 0.23 | 0.33 | 0.19 |
| BreakDancer | DEL | 0.57 | 0.40 | 0.47 | 0.22 |
|  | DUP | 0.03 | 0.00 | 0.00 | 0.00 |
|  | INV | 0.76 | 0.04 | 0.08 | 0.00 |
| MetaSV | DEL | 0.29 | 0.79 | 0.42 | 0.32 |
| *FusorSV* | DEL | 0.60 | 0.65 | 0.62 | 0.43 |
|  | DUP | 0.15 | 0.22 | 0.19 | 0.17 |
|  | INV | 0.50 | 0.44 | 0.45 | 0.40 |

***Additional file 4: Table S1.*** Results of the 1000 iterations of the random validation model that shows the precision (prec), recall (rec), f-score (f1) and Jaccard Similarity (J) averaged across all samples / iterations.

| **Caller** | **SV Type** | **prec** | **rec** | **f1** | **J** |
| --- | --- | --- | --- | --- | --- |
| BreakSeq | DEL | 0.56 | 0.21 | 0.30 | 0.19 |
| cnMOPS | DEL | 0.09 | 0.02 | 0.03 | 0.03 |
|  | DUP | 0.00 | 0.00 | 0.00 | 0.05 |
| CNVnator | DEL | 0.35 | 0.15 | 0.21 | 0.11 |
|  | DUP | 0.22 | 0.03 | 0.05 | 0.11 |
| DELLY | DEL | 0.66 | 0.42 | 0.51 | 0.20 |
|  | DUP | 0.05 | 0.01 | 0.02 | 0.02 |
|  | INV | 0.79 | 0.04 | 0.08 | 0.00 |
| GenomeSTRiP | DEL | 0.38 | 0.42 | 0.40 | 0.24 |
|  | DUP | 0.26 | 0.14 | 0.18 | 0.13 |
| Hydra | DEL | 0.42 | 0.44 | 0.43 | 0.21 |
|  | DUP | 0.02 | 0.01 | 0.01 | 0.00 |
|  | INV | 0.55 | 0.09 | 0.16 | 0.01 |
| LUMPY | DEL | 0.53 | 0.18 | 0.26 | 0.15 |
|  | DUP | 0.04 | 0.02 | 0.03 | 0.02 |
|  | INV | 0.54 | 0.17 | 0.25 | 0.10 |
| BreakDancer | DEL | 0.57 | 0.38 | 0.46 | 0.21 |
|  | DUP | 0.03 | 0.00 | 0.00 | 0.01 |
|  | INV | 0.76 | 0.04 | 0.08 | 0.00 |
| MetaSV | DEL | 0.29 | 0.76 | 0.42 | 0.32 |
| *FusorSV* | DEL | 0.60 | 0.63 | 0.61 | 0.40 |
|  | DUP | 0.15 | 0.20 | 0.17 | 0.15 |
|  | INV | 0.40 | 0.46 | 0.41 | 0.28 |

***Additional file 4: Table S2.*** Results of the 27 samples of the 1000GP shows the precision (prec), recall (rec), f-score (f1) and Jaccard Similarity score (J) averaged across all samples.

| **Deletion** | | | | | | | | |
| --- | --- | --- | --- | --- | --- | --- | --- | --- |
| **Chr** | **Start** | **End** | **Sample** | **PCR Forward Primer** | **PCR Forward Primer** | **PCR Forward Primer** | **ddPCR Forward Primer** | **ddPCR Probe** |
| 1 | 196736209 | 196745258 | NA18525 | TCCCTCAGCCTCTCTTCTCA | CGCTAAGCACTCCTTCTCCA | CTTTTGAGTTAGCTGGGTGTG | AAGCAATCCTTTCACCTCATC | TTGACCCAGGCCTATAGTCCCA |
| 1 | 238852307 | 238853250 | NA19017 | CGCTGCATGGATCAAGGTAT | TGGAGGAAATCCAGAGATGC |  |  |  |
| 1 | 245002401 | 245002802 | HG00419 | GGTGGCTGATGAGAGGAGAG | AGGAGTGGAGGCAGAAGTCA | AATAGGGCCAATTCATCCTT | GAGACAGTTGATTTTCCACCA | CCAGAGGAAAGAAATACGGGCA |
| 2 | 90249467 | 90260301 | NA19017 | GCACATCCTCCTACCCTGAA | CACTGAATCCCTGGCATTTT |  |  |  |
| 2 | 181453907 | 181454631 | NA19239 | CCCATGTAGTTGAACTTATTCCTG | CTGAAAACCATCGCACCAG |  |  |  |
| 3 | 42893225 | 42893983 | NA19239 | AGGGGACACAGGGAGAGACT | ACAGGGCTTTGTGTTTCCAG |  |  |  |
| 3 | 104590687 | 104597787 | NA19239 | CTGGCGAAGGGTCTCTTACA | ATCTGGGGTTTTTGTGTGTG |  |  |  |
| 4 | 16944237 | 16950743 | NA19017 | ACTCCCTTCCTCCCACAAAT | ATCCTGGTGTTTGGGTGATG |  |  |  |
| 4 | 157557363 | 157557837 | NA19238 | TTTGGCCTCAAATTTTCCAC | TGGCTGTGGGAGAAAAGTGT |  |  |  |
| 5 | 9734910 | 9735632 | NA19239 | GGAGATGCAAGAAGCAGAGG | CTGGAGAAGGTGGAAGCAAG |  |  |  |
| 5 | 13416591 | 13422853 | NA12878 | CCTCCAGATTCCAAAGATGC | GGATGGCAAGGAAGACACTC |  |  |  |
| 5 | 49499397 | 49500482 | NA19238 | TCAGCTTCTATAAAAGGAGCGTTTC | GAGCATTCATCCCACAAGGT |  |  |  |
| 5 | 180569233 | 180569978 | NA19239 | AAGCCATTTGGTCTTGGACTT | TGGTTTGAGCCATAAAGTTGG |  |  |  |
| 6 | 72667161 | 72667629 | NA19017 | AAAGGGGAAACAGGACCACT | GCCACGTACCATGACAAATG |  |  |  |
| 7 | 103049 | 104088 | NA19017 | TGCCCTTCAGTTCCTCATTT | TGGCATTGGTTGTAGCACAG |  |  |  |
| 8 | 40289691 | 40296197 | NA19017 | TGCTAATGCCAAGACTGCTG | CCAGGATTCTAAGCCAGGTC |  |  |  |
| 8 | 40289691 | 40296210 | NA19238 | TGCTAATGCCAAGACTGCTG | TCTAAGCCAGGTCATTCATTTG |  |  |  |
| 8 | 65334685 | 65335185 | NA19017 | CAGGGTGATAAGACCATGAGC | TGATGAGGAGAGTGGGGTTC |  |  |  |
| 9 | 115909485 | 115910173 | HG00419 | CAGGGTGATAAGACCATGAGC | TGATGAGGAGAGTGGGGTTC |  |  |  |
| 12 | 64196185 | 64202454 | NA19238 | CAGGGTACTTCTCCGTTGATG | CTTGAAGTGCTGATACCACTGA |  |  |  |
| 12 | 87204804 | 87205597 | NA19239 | CTTGCACCGAGAAATAAATC | CTATGAGTGCCTTGGTGAAC |  |  |  |
| 12 | 92636830 | 92650402 | NA19238 | CATGCCCATCCTGGTATTTT | GCCTGGGCAATGAAGTAAGA |  |  |  |
| 14 | 20315081 | 20315665 | NA19017 | TTTCCGGTAACCCAGACAAC | GCCACAAGCTCAACACACAC |  |  |  |
| 14 | 91959530 | 91960393 | NA19239 | CCCACAAACCCCATTTCTTT | GCCCTTATTTGTGGGTTTGA |  |  |  |
| 15 | 22336853 | 22344093 | NA19238 | GGAGCCCTTCCTTTTTGATT | CCCACCTCCAACTTCTCTTG |  |  |  |
| 16 | 35276844 | 35277808 | NA19017 | AAGAGCTTCGGGCCTATTTT | TGTGTGGTTTTTAGGGGAAC |  |  |  |
| 17 | 10886858 | 10895731 | NA19238 | TCACCCTATCCACAGATGACC | AGAGGTGCTGACTGTGGAAAA | GTTGAGTCCAAAGTGCTTGAA | ATAGAGGTGGCAGAACTTGCT | TCTCACCTTCTCTGTGCAATCCTTC |
| 17 | 37448147 | 37448754 | NA19238 | GACCACAGACATGCATCACC | GTCCAGAAACCCAGCAATGT |  |  |  |
| 18 | 38006374 | 38007000 | NA19239 | TGGGAGTGAAAACTGTGGATG | GCTCAATGGCAACTAGCACTG |  |  |  |
| 18 | 76373726 | 76374274 | NA19239 | TTGAGCATGGTGTCAGGAAG | AGCCCAGTTTCCAACCATTA |  |  |  |
| 22 | 17333036 | 17333646 | NA19239 | GCCATCTCCAACACTGAAGG | GGAGTGACACATTGGCAAAA |  |  |  |
| **Duplication** | | | | | | | | |
| **Chr** | **Start** | **End** | **Sample** | **Control sample** | **ddPCR Forward Primer** | **ddPCR Forward Primer** | **ddPCR Probe** |  |
| 1 | 11051363 | 11053933 | NA12878 | NA10851 & 12878 | GAATAACTGAGCACAGGGACA | GCTGCAGTGTATGGGATAGTG | ATCTTCAGGGGAACCAGCAAGT |  |
| 1 | 17199957 | 17206801 | NA19239 | NA10851 & 12878 | ACAAGGGACAGCTTCAAATG | GAAATAAGCCACTGGTCCAA | TTCAAAGGCTGAGGAATTGC |  |
| 1 | 17199957 | 17214728 | HG00419 | NA10851 & 12878 | ACAAGGGACAGCTTCAAATG | GAAATAAGCCACTGGTCCAA | TTCAAAGGCTGAGGAATTGC |  |
| 1 | 17229408 | 17231701 | HG00419 | NA10851 & 12878 | TGTGGTGGCAAATACCTTTT | CCAAGTGGCTTCTTTTCTGA | CATTTCCCATGTAGTAGAACCTTCTC |  |
| 1 | 17231901 | 17241213 | HG00419 | NA10851 | ACATGTCAGCAGGTAGGTTTG | CTTAAGTTCCACCGACAGTGA | CCTCAGGTTGGGCACATTTTAC |  |
| 1 | 153673146 | 153680779 | NA19625 | NA10851 & 12878 | CCACATCGAAAACAGAGTAGG | GTGCAGTGGTTATTCATAGGC | CCATGCTAATCAGTAATGGGATCC |  |
| 2 | 70016780 | 70021842 | NA19625 | NA10851 & 12878 | TCTATTACCGATGCCTTTCTG | CTGCTTACTCCTGTCAACCAA | TTTTCTCTGAGCTGCAGCCAGA |  |
| 2 | 91785223 | 91810501 | NA19238 | NA10851 & 12878 | TGTCTCATGCATCGTAGGAGT | CTTTCTTCCTGTGTTCATCCA | TGGCTGAATGAATCCATGGATG |  |
| 3 | 99207424 | 99216657 | HG00419 | NA10851 & 12878 | AAAAGACCCAGAATAGCCAAG | GTGGGCATGGATTGATAAAGT | CAAAACTGGAGGAAACACATTACCTG |  |
| 5 | 712901 | 723193 | NA19239 | NA10851 & 12878 | TTCAGAGCCTTCATTTTTGTG | TATCAGCACACATGCAGAAGA | TGCACTGCTGAAAGAATGTTGC |  |
| 5 | 799568 | 807901 | NA19239 | NA10851 & 12878 | GGAAGAGCAACTTCAGTGACA | CTCTTGTTAAAGGCTGGAACC | TTTGGAAAGGGCACAAACTGAT |  |
| 6 | 62184137 | 62189856 | NA19625 | NA10851 & 12878 | AGAGCTAAGGTGAAAAAGCA | GTGAGCACATCACGTAGCC | TCCCAGGATAAAAACTAGAAGGAAGC |  |
| 7 | 78672857 | 78675031 | NA19017 | NA10851 & 12878 | GAAAGCAGATCAGTGGCTTC | CAAAAGTTCCCTTCTGCTACA | AGGGGTGAGGATGAGGTTTTCC |  |
| 8 | 8072301 | 8086086 | NA19625 | NA10851 & 12878 | AACCCTTGCTGTGAATAGGAG | GCAACAAGCTAACAAGCAAGT | CCAAATGCGATCATTATGTTTGC |  |
| 8 | 8072701 | 8086086 | NA19239 | NA10851 & 12878 | AACCCTTGCTGTGAATAGGAG | GCAACAAGCTAACAAGCAAGT | CCAAATGCGATCATTATGTTTGC |  |
| 8 | 47524801 | 47540655 | NA12878 | NA10851 | GTCAAGACATGGAGGGAATG | TCCTGAGCCCTCTTATCCTT | TCCAGGTTCTGTGTTTGGAAGA |  |
| 8 | 47603671 | 47610641 | NA18525 | NA10851 | GGAGATCTGAGACCGGACAG | CTGTCAGTCTGCCCCTAATG | TGGATCCCTGACCCCTGAGTAG |  |
| 10 | 47535982 | 47566352 | NA19239 | NA10851 & 12878 | CACAAACTTCTCCCCATGAT | GGAAGCAGTGGGATTACCTT | CACACATACATACACATCTCCCAGA |  |
| 11 | 58811432 | 58831410 | NA19017 | NA10851 & 12878 | TAGTCTGAAGAATGCCACAGC | CCATCTTCATGTCCTTGCTTA | TGCCACCATGCTGCTTAGG |  |
| 15 | 23216235 | 23227501 | NA18525 | NA10851 & 12878 | CAATCCAGCTTCCTTCTTCTC | TAAGCAGACTTGGACATGGAG | CAGTGCCTTGGATTCAAGGG |  |
| 15 | 89464120 | 89467491 | NA19017 | NA10851 & 12878 | CTCAGAGAACCCCACTTATTTG | AAGGCAAAGAGCAGATAGTCAC | TGGTCACATTCCATGAGCCCTA |  |
| 19 | 27801445 | 27803341 | NA19239 | NA10851 & 12878 | CACCTCACCGACTTGAATCT | CGTAGGAATCAAAGTGCTCA | TCTTTGGATTGAGCAGCTTTGA |  |
| 19 | 37485799 | 37494538 | HG00419 | NA10851 & 12878 | ATAGCTCACCCTTTTGCCTTA | CTTCACAGCAACCCACTTTTA | AGGCAAATAATGTGAAGAAACCCAG |  |
| 20 | 32036970 | 32043186 | NA19017 | NA10851 & 12878 | CCAGTGGTATCAGACAGCAAC | CCTGTGTGTTAACCCTCCTCT | TACAGCAGCAGCAGAGGGACTG |  |
| 21 | 10612780 | 10623200 | NA19625 | NA10851 & 12878 | TGTCTGCCTGAAAAATCACA | ACAAAGAACAGAAGGCATCG | ATAAGCATGCAGGGTCCAGC |  |
| 21 | 37723356 | 37726639 | NA18525 | NA10851 & 12878 | CCTTGAACAGCGCAGTTATTA | CAGGCTCTTAAGGTTCTGAGG | CCCCTCAGTCAAAGATCCACGT |  |
| 22 | 24058444 | 24061701 | NA12878 | NA10851 | AAGGTATCCAACTTCCCAGTG | CCTCGGTCAAATTCTTTCTTC | TAGCAACCTCAATTCTTGCCGC |  |
| **Inversion** | | | | | | | | |
| **Chr** | **Start** | **End** | **Sample** | **Forward Primer A** | **Reverse Primer B** | **Forward Primer C** | **Reverse Primer B** |  |
| 1 | 205178633 | 205178774 | NA12878 | GTTTTTATCTGGGATGGACA | ACCAACTAAATGGAACGGTA | ATAAAAGGAGGGGGATGAC | TGAATAGGTGTGTGAAGTGG |  |
| 2 | 209041654 | 209041825 | NA19238 | CCATTCCCTGAGTAGCAGAA |  |  | ATGGAATTTCCCTATTGTTGC |  |
| 3 | 44741093 | 44742178 | NA19239 | validated by PacBio data and not design primer primers | | | | |
| 3 | 45084291 | 45084454 | NA12878 | AACGAAACTCCCCTACATAAG | CATGCCTACTTCCTACTGAAA | GGACTTAGCCAGGTATGATG | GAGAAAACCCGCCTCTAC |  |
| 5 | 147553039 | 147554607 | NA19017 | ACTTTGTGCGTGAGGTATTT | CACAGTAAGACCCCACCTAA | AGCAATTACCATCTACCCTTC | GAGCTGGTTAATCCCAATAGT |  |
| 8 | 25912314 | 25912455 | NA12878 | CTCCTGAACGACTGGGATTA |  |  | CAAGTTCCCCATATTTCAGC |  |
| 20 | 10788232 | 10789267 | NA19017 | AGTAATTGCACTTTGCTGGT | TTCAGATCCATTCTCCACTC | TCTCTCTGTTGGTGAGGTTT | GTTGAGTGTTTGTCCTGGAT |  |

***Additional file 4: Table S3.*** Novel structure variations detected by *FusorSV* and confirmed by *in vitro* validation.

| ***SVE* ID** | **Caller** |
| --- | --- |
| 35 | BreakSeq |
| 9 | cnMOPS |
| 10 | CNVnator |
| 11 | DELLY |
| 14 | GenomeSTRiP |
| 17 | Hydra |
| 18 | LUMPY |
| 4 | BreakDancer |
| 0 | Truth |
| 1 | MetaSV |
| -1 | *FusorSV* |

***Additional file 4: Table S4.*** *Each tool in SVE has a unique SVE ID.*
