# Supplementary material for: FusorSV: an algorithm for optimally combining data from multiple structural variation detection methods
Source: Genome Biol. 2018 Mar 20;19:38. doi: 10.1186/s13059-018-1404-6 (PMC5859555; doi:10.1186/s13059-018-1404-6)
Supplement: Supplementary file 6 — Reviewer reports and Author’s response to reviewers. (DOCX 34 kb) [file 13059_2018_1404_MOESM6_ESM.docx]

Reviewer 1 report:

**Are the methods appropriate to the aims of the study, are they well described, and are necessary controls included?**

Key details are missing from the methods.

**Are the conclusions adequately supported by the data shown?**

Yes

**Are sufficient details provided to allow replication and comparison with related analyses that may have been performed?**

No. I was unable to run their software.

**Does the method perform better than existing methods (as demonstrated by direct comparison with available methods)?**

Yes

**Is the method likely to be of broad utility? Is any software component easy to install and use?**

It is of broad utility. The software is easy to install, but it does not work.

**Is the paper of broad interest to others in the field, or of outstanding interest to a broad audience of biologists?**

No

**Comments:**

Becker et al. present SVE, a pipeline for running multiple SV calling algorithms, and FusorSV, a method for merging those call sets based on prior training. It has been known for some time that individual SV methods excel for certain SVs, and that several algorithms must be used to get a comprehensive call set. The authors put forward a much-needed framework for discovering how methods should be combined.

While I the method is innovative and would be of broad interest to the community, there are several areas that must be improved. 

1. The method must work. SVE completed (see below) and FusorSV did not. The target BAM was chromosome 21 from the 1000 genomes high-coverage NA12878 sample, and all commands were run on the AWS Ubuntu AMI ami-d15a75c7. Below are the commands I ran:

sudo docker pull timothyjamesbecker/sve

time sudo docker run -v /mnt/local:/data timothyjamesbecker/sve /software/SVE/scripts/variant_processor.py -r /data/g1k_v37_decoy.fa -b /data/NA12878.21.bam -o /data/vcfs/ -s breakdancer,gatk_haplo,delly,lumpy,cnvnator 2> run.stderr 1> run.stdout real 252m35.868s user 0m0.024s sys 0m0.036s

time sudo docker run -v /mnt/local:/data timothyjamesbecker/sve /software/FusorSV/FusorSV.py
container_linux.go:247: starting container process caused "exec: \"/software/FusorSV/FusorSV.py\": stat /software/FusorSV/FusorSV.py: no such file or directory"
docker: Error response from daemon: invalid header field value "oci runtime error: container_linux.go:247: starting container process caused \"exec: \\\"/software/FusorSV/FusorSV.py\\\": stat /software/FusorSV/FusorSV.py: no such file or directory\"\n".

While SVE ran, it produced +16MB of output to STDOUT with many apparent errors. 
When it is unclear if a tool was fully or even partially successful then its usability is low. A docker container that includes all of these methods is a great idea and will be very useful to the community. If the authors can make SVE more robust it will likely gain broad usage.

Reviewer 2 report:

**Are the methods appropriate to the aims of the study, are they well described, and are necessary controls included?**

Yes

**Are the conclusions adequately supported by the data shown?**

Yes

**Are sufficient details provided to allow replication and comparison with related analyses that may have been performed?**

Yes. Need to classify access to the 27 trio data for reproducability

**Does the method perform better than existing methods (as demonstrated by direct comparison with available methods)?**

Yes

**Is the method likely to be of broad utility? Is any software component easy to install and use?**

Not necessarily. It requires running 6-7 front-end callers, which may not be very practical. Also, the experiments are carried out in specific settings (deep trio data), which may not be generalizable to other settings.

**Is the paper of broad interest to others in the field, or of outstanding interest to a broad audience of biologists?**

No

**Comments:**

There are key details missing from the main paper. 

How is the model used? The "Application of the Model" is one sentence. From the previous training section I know that every possible combination of methods is scored, but I do not know how that score is used. What about the corner cases when an SV is on the edge between to SV bins? 

How are two SVs determined to be the "same" SV? This issue is avoided to some extent in the training by using Jaccard, but if you are going merge calls from different methods there will either be merging or redundancy. 

Once two SVs are determined to be the "same", how are they merged? 
I know that much of this is described precisely in the supplement and I appreciate the rigor, but simple and straightforward prose of these critical details must be added to the main text. In addition to how these steps work, some analysis of the merging would be helpful since in many cases the merged SV is quite different than the source SVs. How do you avoid merging a chain of transitively related SVs? Is it possible that the merged SV ends up in a different size bin?

3. The most interesting result, which combination of methods gives the most complete call set for each SV type/size, is not reported. The number of genomes that are considered in SV experiments is growing quickly, and most labs will not have the resources or patience to run 6 different methods for every sample if running 2 methods will be nearly as good. I strongly suggest that the authors add a section to the paper that explores these results in detail. For example, the authors went through the trouble of scoring the power set of methods, why not do so analysis of that set? You can plot well do the top 100 perform, evaluate methods based on their rank within the power set, etc. 

Reviewer 3 report:

**Are the methods appropriate to the aims of the study, are they well described, and are necessary controls included?**

Yes

**Are the conclusions adequately supported by the data shown?**

Yes

**Are sufficient details provided to allow replication and comparison with related analyses that may have been performed?**

Yes.

**Does the method perform better than existing methods (as demonstrated by direct comparison with available methods)?**

Yes

**Is the method likely to be of broad utility? Is any software component easy to install and use?**

See also below (Is the paper of broad interest to others in the field, or of outstanding interest to a broad audience of biologists?). The method FusorSV compares very favourably with MetaSV, a tool with a similar rationale, and thus I believe will be of broad utility.

**Is the paper of broad interest to others in the field, or of outstanding interest to a broad audience of biologists?**

Yes: Structural Variations (SVs) are among the most significant determinants of human genetic diversity, leading to most nucleotide differences amongst the genomes of unrelated individuals. Yet, SVs are known to be much more difficult to infer than other classes of genetic variation (namely SNPs). The structural variation field is well-aware of the issue that no single SV calling algorithm enables identifying all SV classes or (within an SV class) SVs across all size ranges, which is why most laboratories use ensembles of SV callers. It is, however, not immediately clear how the output of different SV callers can be most meaningfully and successfully integrated, as the simple use of callset unions is likely to lead to an increased rate of false positives while the use of overlapping calls (such supported by multiple callers) is prone to result in false negatives. With FusorSV the authors provide a useful and elegantly developed solution in this regard, which I believe a number of groups are likely to implement to enable them to run a variety of SV callers in personal genome analyses.

**Comments:**

Structural Variations (SVs) are among the most significant determinants of human genetic diversity, leading to most nucleotide differences amongst the genomes of unrelated individuals. Yet, SVs are known to be much more difficult to infer than other classes of genetic variation (namely SNPs). The structural variation field is well-aware of the issue that no single SV calling algorithm enables identifying all SV classes or (within an SV class) SVs across all size ranges, which is why most laboratories use ensembles of SV callers. It is, however, not immediately clear how the output of different SV callers can be most meaningfully and successfully integrated, as the simple use of callset unions is likely to lead to an increased rate of false positives while the use of overlapping calls (such supported by multiple callers) is prone to result in false negatives.

To this end, this manuscript presents and approach, FusorSV, that uses data mining to assess the performance and merge callsets from an ensemble of SV-calling algorithms to arrive at an extended high quality set of SVs for a 27 deeply sequenced genomes that are part of the 1000 Genomes Project (1000GP) set of samples. The authors additionally, to facilitate application of FusorSV, present Structural Variation Engine (SVE), a pipeline that enables launching eight popular SV tools with little manual intervention. The authors identify over 800 SVs that were not identified in the 1000GP, and show based on experimental validation that these contain a high rate of true calls, indicating a high quality of predictions. This manuscript is overall well-written and presents a method that is likely going to be of utility for calling SVs beyond data from the 1000 Genomes Project, such as in more biomedically oriented studies (e.g. TOPMED). With this in mind, it would be important that the authors reassure themselves they have not over-trained their method based on the genomes they chose to analyse here (27 genomes sequenced deeply by the Broad institute in the course of the 1000GP). In this regard, it would be most helpful, and indeed an important confirmation of the work presented here, if another dataset could be investigated by the authors -- at the very least for the measures precision and recall. The authors could, for example, use low-coverage data from the 1000 Genomes Project (sequencing data largely generated with a different Illumina protocol, if I am not mistaken) to make this independent assessment - or alternatively they could choose to utilize a different dataset for this. Doing so would help the authors ensure wide applicability/utility of FusorSV.

As an additional question, can the authors please clarify whether the 800 novel SVs inferred by their method, which had been overlooked by the 1000 genomes project, had all been analyzed at the level of high-coverage data by the 1000GP investigators (or where these genomes analyzed at the level of low-coverage data by the 1000GP investigators)? I found this part confusing to be frank, as I think there was no released callset for the 27 high coverage genomes by the 1000GP, is that right? (If not, which file did the authors use, to compute the delta of 800 novel variants?) Or are these 800 novel variants novel largely because the authors compare the outcome of FusorSV on high coverage genomes to 1000GP work performed on low coverage? (if so, wouldn't that be an "apples-to-oranges" comparison?)

Author response to reviewers

We would like to thank you for considering our manuscript entitled “FusorSV: An algorithm for optimally combining data from multiple structural variation detection methods” by Becker et al. for publication in the journal Genome Biology.

We would also like to thank the reviewers for providing very informative critique of the manuscript. We have addressed all the points raised by the reviewers either by adding more information to the manuscript and/or provided detailed point-by-point response below. We have since designed a fresh docker container that includes the FusorSV/SVE method and the framework has been tested and verified to be working correctly. All of the code for the manuscript is also made available on Github with GNU General Public License.

We hope that you would find our response and revised manuscript appropriate for

publication in your journal.

Reviewer #1: Becker et al. present SVE, a pipeline for running multiple SV calling algorithms, and FusorSV, a method for merging those call sets based on prior training.

It has been known for some time that individual SV methods excel for certain SVs, and that several algorithms must be used to get a comprehensive call set. The authors put forward a much-needed framework for discovering how methods should be combined.

While I the method is innovative and would be of broad interest to the community, there are several areas that must be improved.

RESPONSE: We would like to thank the reviewer for their very critical review of the manuscript. We also acknowledge that FusorSV provides a much needed framework around the problem of integrating callsets from multiple SV algorithms. We have considered all of the points raised by the reviewer and provided more information in the manuscript as well as below. Please see the following for a point-by-point response.

R1Q1: 1. The method must work. SVE completed (see below) and FusorSV did not. The target BAM was chromosome 21 from the 1000 genomes high-coverage NA12878 sample, and all commands were run on the AWS Ubuntu AMI ami-d15a75c7. Below are the commands I ran:

sudo docker pull timothyjamesbecker/sve

time sudo docker run -v /mnt/local:/data timothyjamesbecker/sve

/software/SVE/scripts/variant_processor.py -r /data/g1k_v37_decoy.fa -b

/data/NA12878.21.bam -o /data/vcfs/ -s breakdancer,gatk_haplo,delly,lumpy,cnvnator

2> run.stderr 1> run.stdout

real 252m35.868s

user 0m0.024s

sys 0m0.036s

time sudo docker run -v /mnt/local:/data timothyjamesbecker/sve

/software/FusorSV/FusorSV.py

container_linux.go:247: starting container process caused "exec:

\"/software/FusorSV/FusorSV.py\": stat /software/FusorSV/FusorSV.py: no such file or

directory"

docker: Error response from daemon: invalid header field value "oci runtime error:

container_linux.go:247: starting container process caused \"exec:

\\\"/software/FusorSV/FusorSV.py\\\": stat /software/FusorSV/FusorSV.py: no such file

or directory\"\n".

While SVE ran, it produced +16MB of output to STDOUT with many apparent errors.

When it is unclear if a tool was fully or even partially successful then its usability is low. A docker container that includes all of these methods is a great idea and will be very useful to the community. If the authors can make SVE more robust it will likely gain broad usage.”

RESPONSE: We apologize to the reviewer that we did not make the Docker image consistent with the updates on github and provided an incorrect legacy Docker image.

The issue has been fixed. We provide a Dockerfile at https://github.com/TheJacksonLaboratory/SVE/blob/master/Dockerfile for users to build an image from scratch or the user can pull a pre-built image from https://hub.docker.com/r/wanpinglee/sve/. The release version is 0.1.0, which is current for both github and dockerhub versions. All features in the docker image have also been tested and verified to be working correctly.

R1Q2: How is the model used? The "Application of the Model" is one sentence. From the previous training section I know that every possible combination of methods is scored, but I do not know how that score is used. What about the corner cases when an SV is on the edge between to SV bins?

RESPONSE: We have added the required language to the manuscript (highlighted using track changes under FusorSV overview, training and discovery sections) that describes how the trained model is used. To clarify, we use disjoint bins based on SV types and sizes and SVs would always be assigned to only one bin.

R1Q3: How are two SVs determined to be the "same" SV? This issue is avoided to some extent in the training by using Jaccard, but if you are going merge calls from different methods there will either be merging or redundancy. Once two SVs are determined to be the "same", how are they merged?

I know that much of this is described precisely in the supplement and I appreciate the rigor, but simple and straightforward prose of these critical details must be added to the main text. In addition to how these steps work, some analysis of the merging would be helpful since in many cases the merged SV is quite different than the source SVs. How do you avoid merging a chain of transitively related SVs? Is it possible that the merged SV ends up in a different size bin?

RESPONSE: We have added more information to the manuscript in several places (in the sections titled FusorSV training and discovery) to help readers understand the merging process better. We acknowledge that when detecting SVs using paired-end (PE) based approach transitively related chains could emerge especially in the repetitive regions of the genome. However this is not true for read-depth (RD) based callers such as GenomeSTRiP, CNVnator, cnMOPS that do not suffer from this issue.

Using our method with a nice mixture of SV calling algorithms including RD and PE methods helps us to avoid this to a large extent.

R1Q4: 3. The most interesting result, which combination of methods gives the most complete call set for each SV type/size, is not reported. The number of genomes that are considered in SV experiments is growing quickly, and most labs will not have the resources or patience to run 6 different methods for every sample if running 2 methods will be nearly as good. I strongly suggest that the authors add a section to the paper that explores these results in detail. For example, the authors went through the trouble of scoring the power set of methods, why not do so analysis of that set? You can plot well do the top 100 perform, evaluate methods based on their rank within the power set, etc.

RESPONSE: We agree with the reviewer that the most significant result here is the combination of methods that gives the best resulting SV call set. However we would like to point out that all callers included in our ensemble make some contribution to the final result. To provide the information about the high performing combination of SV callers, we have added an additional supplemental figure and data table (supplemental figure 6 and supplementary table 5) and text to the manuscript (changes are highlighted) that highlight the training and scoring of various combinations of callers and their selection to derive a final result.

We included MetaSV in our comparisons to show how FusorSV compares to a straightforward combination of caller approach (supported by at least two callers). We explored various combinations of the eight callers and some of the combinations did perform better than MetaSV. The fusion method employed in FusorSV is demonstrably superior to those unpublished combinatorial methods.

Reviewer #2: The work FusorSV presented a method that combines SV calls from multiple SV callers to generate a better result. The method essentially performed linear discriminate analysis in a series of SV size/type bins, using the “high confidence” 1000 phase 3 SVs as the training set and assuming that the complementary properties of the algorithms can be captured by the linear decision rules and can be generalized to new data. Overall, the work has some practical value to the ongoing SV detection analysis and the 1000G/the deep trio data are valuable for performing such kind of investigation. Encouragingly, the experimental validation results look good.

RESPONSE: We would like to thank the reviewer for the detailed and informative comments.

A few questions that I think should be addressed:

R2Q1: 1) Did the authors consider features other than SV type and sizes? For example, sequence context and repeat annotations. Why and why not?

RESPONSE: We used SV types and sizes as discriminating features since they have the biggest impact on the performance (discrimination) of the various SV calling algorithms. We further tried gene/nongene and repeat/non-repeat as features however did not have the same level of “discrimination” to use them as with types and sizes.

However some of the SV callers such as Delly themselves take repeat annotations into account when calling SVs.

This question postulates a further question of using arbitrary features with deep learning or PCA/SVD methods directly. While such an approach might lead to a very nice fit on the training data, we cannot guarantee if it would be generalizable to new data.

By using simple and explainable features that work well, the result remains in a tangible form that can be discussed such as SV X has a 0.5 expectation of being correct given the g1kP3 model. It remains an open research question as to what other features can be used to provide better accuracy in SV calling and something that we would like to explore in the future.

R2Q2: 2) How was the final call set produced? Were they selected from the test sets in the 1,000 rounds of 18/9 random splitting experiments? It is important to ensure that results/models are generalizable to other settings.

RESPONSE: We produced the final call set by training a model with all 27 high coverage samples and then running those SV callsets as inputs in discovery mode.

The fact that this approach produces new novel calls that can be validated (with a high experimental success rate) even though we did not use any form of instance-based learning to fit the model implies fusorSV is a generalizable method

R2Q3: 3) Did the algorithm achieve similar accuracy in all 27 samples? Or performed better on high coverage samples?

RESPONSE: All of the 27 samples had high coverage data sequenced to about the same ~50X coverage using Illumina PCR free sequencing protocol. Figure 3B shows the performance of all the single callers as well as FusorSV. As it can be seen, the convex hulls of DELETION and DUPLICATIONS for FusorSV are tight, indicating that all the samples performed similarly. However, the convex hull of INVERSIONS does not show the same pattern, indicating a variance in the various samples. This could be because the model is not trained optimally due to the lack of enough training inversions

in the truth set (1kgp3).

We have also added a new supplemental figure 7, Caller performance by sample. The figure includes three plots per sample, that show the performance (precision vs recall) of the individual callers, including FusorSV separated by DELETIONS/DUPLICATIONS and INVERSIONS.

R2Q4: 4) The qualify of figures needs improvement. It is difficult to distinguish the colors.

RESPONSE: We have replaced Fig 3 and Fig 4 with higher quality versions.

Reviewer #3: Structural Variations (SVs) are among the most significant determinants of human genetic diversity, leading to most nucleotide differences amongst the genomes of unrelated individuals. Yet, SVs are known to be much more difficult to infer than other classes of genetic variation (namely SNPs). The structural variation field is well-aware of the issue that no single SV calling algorithm enables identifying all SV classes or (within an SV class) SVs across all size ranges, which is why most laboratories use ensembles of SV callers. It is, however, not immediately clear how the output of different SV callers can be most meaningfully and successfully integrated, as the simple use of callset unions is likely to lead to an increased rate of false positives while the use of overlapping calls (such supported by multiple callers) is prone to result in false negatives.

To this end, this manuscript presents and approach, FusorSV, that uses data mining to assess the performance and merge callsets from an ensemble of SV-calling algorithms to arrive at an extended high quality set of SVs for a 27 deeply sequenced genomes that are part of the 1000 Genomes Project (1000GP) set of samples. The authors additionally, to facilitate application of FusorSV, present Structural Variation Engine (SVE), a pipeline that enables launching eight popular SV tools with little manual intervention. The authors identify over 800 SVs that were not identified in the 1000GP, and show based on experimental validation that these contain a high rate of true calls, indicating a high quality of predictions. This manuscript is overall well-written and presents a method that is likely going to be of utility for calling SVs beyond data from the 1000 Genomes Project, such as in more biomedically oriented studies (e.g. TOPMED).

RESPONSE: We thank the reviewer for the in-depth review of the manuscript and agree that there is currently a need in the field for a method like FusorSV that allows for intelligent merging of multiple SV calles. We are actively keeping abreast of highquality publically available genomic data and will be providing additional FusorSV models. Before we can work with disease-based models, we thought it prudent to establish a healthy germline model.

R3Q1: With this in mind, it would be important that the authors reassure themselves they have not over-trained their method based on the genomes they chose to analyse here (27 genomes sequenced deeply by the Broad institute in the course of the 1000GP). In this regard, it would be most helpful, and indeed an important confirmation of the work presented here, if another dataset could be investigated by the authors – at the very least for the measures precision and recall. The authors could, for example, use low-coverage data from the 1000 Genomes Project (sequencing data largely generated with a different Illumina protocol, if I am not mistaken) to make this independent assessment - or alternatively they could choose to utilize a different dataset for this. Doing so would help the authors ensure wide applicability/utility of FusorSV.

RESPONSE: We acknowledge that FusorSV does rely on training and tries to best match the input truth set. Which is why the selection of the truth set does become critical in training a good model. At the current stage, the 1000 Genomes Project Phase 3 call set remains the best available SV call set that uses short read Illumina data. We hope to train a new SV calling model using the multi-platform SV call set that would soon be produced by the Human Genome Structural Variation consortium

(HGSV).

However, we would also like to point out that FusorSV does produce novel calls that were not present in the 1000 Genomes Project Phase 3 call set and with a very high experimental validation rate (~85% across all SV types). This implies that FusorSV does learn what constitutes a good mix of input SV call sets and uses this to produce an SV call set that exceeds the training input. As suggested by the reviewer, we did build a model using 100 low coverage samples as well, and still retain ~20% of the novel calls as compared to the 1000 Genomes Project Phase 3 call set.

R3Q2: As an additional question, can the authors please clarify whether the 800 novel SVs inferred by their method, which had been overlooked by the 1000 genomes project, had all been analyzed at the level of high-coverage data by the 1000GP investigators (or where these genomes analyzed at the level of low-coverage data by the 1000GP investigators)? I found this part confusing to be frank, as I think there was no released callset for the 27 high coverage genomes by the 1000GP, is that right? (If not, which file did the authors use, to compute the delta of 800 novel variants?) Or are these 800 novel variants novel largely because the authors compare the outcome of FusorSV on high coverage genomes to 1000GP work performed on low coverage? (if so, wouldn't that be an "apples-to-oranges" comparison?)

RESPONSE: Yes, the 800 novel SVs inferred by FusorSV were using a training model devised using the low coverage data (~5-6X coverage on average) as the truth set to analyze samples that were higher coverage (~50X coverage on average). We agree with the reviewer that this does seem like an “apples-to-oranges” comparison. However we would also like to argue that there was enough truth available in the low coverage 1000 Genomes Project Phase 3 call set for us to devise a training model that works well. This is exemplified by our very high experimental validation rate (~85%) for the novel SVs found. We agree these SVs were probably missed by the 1000 Genomes Project because of the lower coverage, and make no claims to the deficiency in the quality of that call set vis a vis FusorSV output. We simply use it to highlight that the training model, when applied in a blind manner to whole genome sequencing data, FusorSV does discover bonafide SVs that were not included in the training.
